# Prenatal cannabis exposure is associated with alterations in offspring DNA methylation at genes involved in neurodevelopment, across the life course

**DOI:** 10.1038/s41380-024-02752-w

**Published:** 2024-09-14

**Authors:** Alexandra J. Noble, Alex T. Adams, Jack Satsangi, Joseph M. Boden, Amy J. Osborne

**Affiliations:** 1 Translational Gastroenterology Unit, Nuffield Department of Experimental Medicine, University of Oxford, Oxford, UK; 2 Biomedical Research Centre, University of Oxford, Oxford, UK; 3 Christchurch Health and Development Study, Department of Psychological Medicine, University of Otago Christchurch, Christchurch, New Zealand; 4 School of Biological Sciences, University of Canterbury, Christchurch, New Zealand

**Keywords:** Genetics, Psychiatric disorders

## Abstract

Prenatal cannabis exposure (PCE) is of increasing concern globally, due to the potential impact on offspring neurodevelopment, and its association with childhood and adolescent brain development and cognitive function. However, there is currently a lack of research addressing the molecular impact of PCE, that may help to clarify the association between PCE and neurodevelopment. To address this knowledge gap, here we present epigenome-wide association study data across multiple time points, examining the effect of PCE and co-exposure with tobacco using two longitudinal studies, the Avon Longitudinal Study of Parents and Children (ALSPAC) and the Christchurch Health and Development Study (CHDS) at birth (0 y), 7 y and 15–17 y (ALSPAC), and ~27 y (CHDS). Our findings reveal genome-wide significant DNA methylation differences in offspring at 0 y, 7 y, 15–17 y, and 27 y associated with PCE alone, and co-exposure with tobacco. Importantly, we identified significantly differentially methylated CpG sites within the genes *LZTS2, NPSR1, NT5E*, *CRIP2, DOCK8, COQ5*, and *LRP5* that are shared between different time points throughout development in offspring. Notably, functional pathway analysis showed enrichment for differential DNA methylation in neurodevelopment, neurotransmission, and neuronal structure pathways, and this was consistent across all timepoints in both cohorts. Given the increasing volume of epidemiological evidence that suggests a link between PCE and adverse neurodevelopmental outcomes in exposed offspring, this work highlights the need for further investigation into PCE, particularly in larger cohorts.

## Introduction

The use of cannabis during pregnancy has increased in recent years [1–4], becoming the most commonly used drug, excluding alcohol and tobacco, among pregnant women in the US [5, 6]; recent estimates suggest an increase from 3.4% in 2017 to 7.0% in 2022 [4, 7] with these figures still rising post COVID-19 pandemic [8]. The exponential increase in frequency is likely to be a consequence of legalisation and decriminalisation of cannabis [9–12], which may have decreased the public perception of any risk associated with maternal cannabis consumption during pregnancy [13–15]. However, the limited empirical research on prenatal cannabis exposure (PCE) has resulted in a lack of clarity and understanding regarding any potential risks and long-term harm to exposed individuals [16–18].

Recent epidemiological studies indicate an impact of PCE on neonatal health and neurodevelopment [18]. Specifically, PCE is associated with physical neonatal health metrics such as birth weight, birth timing and infant growth restriction [2, 19–21] but also traits related to childhood and adolescent neurodevelopment and brain function, for example, higher-order executive function [22, 23], and psychosocial and neurobehavioural traits such as psychopathy [24] and behavioural development [25]. Furthermore, PCE has observed impacts on Conduct Problem (CP) phenotypes such as externalising behaviours [26], learning disorders, and autism [27], and may increase the risk of neurodevelopmental and psychiatric disorders such as depression, anxiety, attention deficit and psychosis/schizophrenia in adulthood [28]. While these studies are associative, they warrant further investigation to clarify the risk profile of PCE. This is because PCE has the potential to directly impact offspring neurodevelopment; cannabis can cross the placenta [29], and can therefore impact the developing neonatal endocannabinoid system (ECS) [22]. The ECS is integral for correct prenatal neurodevelopment, with roles in neurite outgrowth, migration, and axon growth and guidance [30], as well as synaptic plasticity [31, 32], neuronal specification [33] and immune regulation [34]. Thus, given the central role of the ECS in brain development [22], any interference with this system via PCE may disrupt ECS signalling at critical stages of development, potentially altering structural and functional neurodevelopment [35], brain growth [36], and nervous system functioning in later life [22]. Consequently, because neurodevelopmental disorders and diseases have a complex aetiology, molecular evidence is required to be able to determine whether PCE is implicated in neurodevelopmental outcomes.

Neurodevelopmental phenotypes are often mediated at the molecular level by environmentally-induced genetic and epigenetic change [37–41], such as changes to DNA methylation (an epigenetic modification that is sometimes associated with gene expression levels [42–45]). Indeed, targeted studies from animal models indicate that PCE can impact DNA methylation in the genomes of exposed offspring, e.g. refs. [46–49]. Further studies show an impact of cannabis on the methylome of adult cannabis users, at genes involved in neuronal signalling [50, 51]. Additionally, cannabis exposure is associated with differential methylation at genes enriched for cardiogenesis and neurodevelopment in sperm [52, 53]. Overall, large-scale studies of PCE on human populations, while starting to emerge (e.g. refs. [23, 54]) are still scant, and thus the true nature of the relationship between PCE and neurodevelopment is still not fully understood. Consequently, the scientific and medical communities are in agreement that there is an urgent need for more evidence and research into the impacts of prenatal cannabis exposure on infant neurodevelopment.

Thus, to investigate the molecular impact of PCE, here we assessed DNA from two independent longitudinal studies: i) the Avon Longitudinal Study of Parents and Children (ALSPAC, United Kingdom), and; ii) the Christchurch Health and Development Study (CHDS, New Zealand). Using DNA methylation arrays (Illumina Infinium Human Methylation 450 K BeadChip [ALSPAC] and Illumina Infinium MethylationEPIC BeadChip [CHDS]) over the lifecourse (birth, 7 y and 15 y [ALSPAC] and 27 y [CHDS]) we performed epigenome-wide association studies (EWAS) to investigate the effect of PCE on genome-wide DNA methylation patterns, to explore the hypothesis that PCE will be associated with differential DNA methylation at genes and in pathways that have roles in neurodevelopment and neurodevelopmental disease. The findings from this study will contribute to our knowledge around the potential for PCE to influence neurodevelopmental outcomes.

## Methods

### Avon Longitudinal Study of Parents and Children cohort characteristics

Pregnant women resident in Avon, UK with expected dates of delivery between 1st April 1991 and 31st December 1992 were invited to take part in the study [55, 56]. 20,248 pregnancies were identified as being eligible and the initial number of pregnancies enroled was 14,541. Data is available for 13,988 children who were alive at 1 year of age. A subset of individuals from the Accessible Resource for Integrated Epigenomics Studies (ARIES [57]) at age 0 (*N* = 858), 7 y (*N* = 924), and 15 y (*N* = 922) were assessed for PCE (Table 1). As a longitudinal study, each time point represents the same group of patients, re-sampled at three different times. PCE was characterised based on the self-reported answer to smoked cannabis during pregnancy. Exposure was defined as mothers that responded with ‘every day’ use, 2–4 times per week, <once per week, and once per week. Non-exposed was defined as mothers that responded with ‘not at all’. Ethical approval for the study was obtained from the ALSPAC Ethics and Law Committee and the Local Research Ethics Committees. Informed consent for the use of data collected via questionnaires and clinics was obtained from participants following the recommendations of the ALSPAC Ethics and Law Committee at the time. Please note that the study website contains details of all the data that is available through a fully searchable data dictionary and variable search tool (http://www.bristol.ac.uk/alspac/researchers/our-data/). Consent for biological samples has been collected in accordance with the Human Tissue Act (2004).

**Table 1:** Cohort characteristics for ALSPAC and CHDS.

Cohort	Age (y)	*N*	Sample	Female (%)	PCE	PCTE	PTE	Tobacco smoking	Cannabis user
***ALSPAC***									
Timepoint 1	0	858	Cord blood	448 (52.21%)	10 (1.51%)	30 (3.14%)	168 (19.58%)		
Timepoint 2	7	924	Whole blood	473 (51.19%)	9 (1.08%)	22 (3.46%)	184 (19.91%)		
Timepoint 3	15-17	922	Whole blood	484 (52.49%)	11 (1.19%)	21 (3.57%)	182 (19.73%)		
***CHDS***									
Timepoint 1	~27	98	Whole blood	27 (27.5%)	4 (4.08%)	13 (13.2%)	55 (56.1%)	28 (28.5%)	37 (37.5%)

*CHDS* participants (27 y) were also classified for personal tobacco or cannabis usage as adults.

*PCE* prenatal cannabis exposure controlled for prenatal tobacco exposure, *PCTE* prenatal cannabis exposure with tobacco co-exposure, *PTE* prenatal tobacco exposure only.

### Christchurch Health and Development Study cohort characteristics

The CHDS includes individuals who have been studied on 24 occasions from birth to age of 40 (*N* = 904 at age 30), with blood collected at approximately age 27. A subset of *N* = 98 individuals for whom blood samples were available are included in the current study (Table 1). Of these 98 participants, *N* = 13 were prenatally exposed to cannabis, either with or without co-exposure to tobacco, with *N* = 85 serving as unexposed controls (neither prenatal cannabis nor prenatal tobacco exposure). Usage data was derived from self-reports from mothers at their birth interviews (undertaken in 1977). Tobacco consumption was measured via four questions: How many cigarettes per day did you smoke a) before you were pregnant; b) during the first three months of pregnancy; c) during the second three months of pregnancy; and d) during the last three months of pregnancy. The answers for each trimester were summed to create a measure of the average number of cigarettes smoked during each trimester. Cannabis exposure was assessed via the following self-report question: During pregnancy, did you take any of the following drugs? (y/n response option), with option a) cannabis. Mode of cannabis consumption in this cohort was via smoking, for all participants. All aspects of the study were approved by the Southern Health and Disability Ethics Committee, under application number CTB/04/11/234/AM10 “Collection of DNA in the Christchurch Health and Development Study”, and the CHDS ethics approval covering the collection of cannabis use: “16/STH/188/AM03 The Christchurch Health and Development Study 40 Year Follow-up”.

### DNA extraction and genome-wide methylation profiling for CHDS

DNA was extracted from whole blood samples using the Kingfisher Flex System (Thermo Scientific, Waltham, MA USA), as per the published protocols. DNA was quantified via NanoDrop^TM^ (Thermo Scientific, Waltham, MA USA) and standardised to 100 ng/μl. Equimolar amounts were shipped to the Australian Genomics Research Facility (AGRF, Melbourne, VIC, Australia) for processing via the Infinium® Methylation EPIC BeadChip (Illumina, San Diego, CA USA). The arrays were conducted in groups over four batches (2016, 2017, 2020 and 2022). Analysis was carried out in R statistical software (Version 3.5.2) [58].

### Data analysis for CHDS

Quality control checks were performed on the raw data: firstly, sex chromosomes and a total of 90 failed probes (detection *P* value of <0.01 in at least 50% of samples) were excluded from the analysis. Additionally, CpG sites with adjacent single nucleotide variants or that did not map to a single location in the genome were also excluded [59], removing a total of 195,354 probes from the final CHDS analysis. Functional normalisation was performed and was inspected using beta density distribution plots and multi-dimensional scaling of the 5000 most variable CpG sites. Both slide and array positions were corrected for using ComBat (as implemented by ChAMP [60]) to correct for the batch effects between sampling (four batches). Cell proportions were estimated using the Housman algorithm [61].

### DNA extractions and analysis for ALSPAC

DNA samples were extracted from cord blood on delivery, and from peripheral blood samples at two time points in childhood (7 y and 15–17 y) following established methods [62], and methylation detection was undertaken using the Illumina Infinium Human Methylation450 BeadChip. Data was available post-normalisation and with estimated cell proportions with the Houseman algorithm [61] for the peripheral blood and the cord blood cell type reference for cord blood samples [63].

### EWAS analysis

To partition the data for epigenome-wide analysis, data were separated into several analysis cohorts, by cohort origin (ALSPAC or CHDS), and by age. Specifically, for ALSPAC data, we undertook EWAS at three time points (Table 1); birth, where *N* = 30 case participants were identified, comprised of *N* = 10 exposed to cannabis prenatally, “PCE”, plus *N* = 20 exposed to cannabis and tobacco prenatally, “PCTE”, vs *N* = 654 controls (neither prenatal cannabis nor tobacco exposure); at 7 y old, where *N* = 31 case participants were identified, comprised of *N* = 9 PCE and *N* = 22 PCTE, vs. *N* = 700 controls, and at 15 y where *N* = 32 case participants were identified, comprised of *N* = 11 PCE and *N* = 21 PCTE, vs. *N* = 701 controls. Overall there was missing data on PCE status at each of the time points (*N* = 30, cord blood; *N* = 31, 7 y; *N* = 32, 15 y) and these individuals were removed from further analysis. For CHDS data, we undertook EWAS using *N* = 13 individuals exposed to either cannabis only (“PCE”) or cannabis and tobacco (“PCTE”) prenatally vs. *N* = 85 controls (Table 1).

ALSPAC EWAS analyses were controlled for prenatal tobacco exposure, but this was not possible for CHDS due to small sample sizes. Therefore data for ALSPAC are presented as PCE, corrected for prenatal tobacco exposure, while CHDS data are presented as prenatal cannabis and tobacco exposure, PCTE. To overcome the limitation of a small number of PCE individuals in CHDS, which may prohibit direct comparison between ALSPAC and CHDS datasets, we also present data from EWAS of individuals prenatally exposed to tobacco only (“PTE”, *N* = 55, Table 1) for CHDS, in order to distinguish between cannabis-specific vs. tobacco-associated differential methylation in the CHDS cohort.

The effects of PCE (ALSAPC) and PCTE (CHDS) exposure on methylation were analysed by linear regression with an empirical Bayes correction (limma) for: prenatal tobacco exposure (for PCE/ALSPAC only) (bivariate), adult tobacco status (bivariate), adult cannabis use (bivariate), sex (bivariate), the first principal component of the estimated cell proportions (CD8+, CD4+, granulocytes, B cells, monocytes, natural killer) as covariates. A linear regression model was then fitted to the data with the Q-Q plots of the residuals used to generate lambda values to assess for over-inflation. A similar linear regression model was used to assess prenatal tobacco exposure (PTE, CHDS only), however, the variable prenatal cannabis exposure (bivariate) was corrected for, along with adult tobacco smoking status, sex, and the first principal component of the cell type proportions. Top tables of differentially methylated CpG sites, which were corrected for multiple testing using Benjamini-Hochberg (BH) false discovery rate (FDR), were generated for all models. Differentially methylated CpG sites that were intergenic were matched to the nearest neighbouring genes in Hg19 using Granges [64]. The package ggplot2 (Version 3.3.2) was used to construct all graphs [65].

### Gene ontology

We performed gene ontology enrichment analyses to determine which pathways (biological process, BP; molecular function, MF; cellular component, CC) were significantly enriched in EWAS data, at 0 y, 7 y, and 15–17 y (ALSPAC) and at 27 y (CHDS). The top 5000 differentially methylated loci in response to PCE (ALSPAC) or PCTE (CHDS; nominal *P* < 0.01) were selected and submitted to the EWAS Toolkit [66]. ALSPAC samples were run against a 450 K array background, and CHDS samples were run against the EPIC/850 K array background.

## Results

### Genome wide methylation analysis of prenatal cannabis exposure in ALSPAC cohort at 0 y, 7 y, and 15–17 y

Individual EWAS were performed for all three-time points in the ALSPAC cohort for PCE. With Benjamini–Hochberg (BH) false discovery rate (FDR) correction for multiple testing, 104 CpG sites were significantly differentially methylated (*P* adj < 0.05) at 0 y (cord blood, Table 2a, Supplementary Table 1 and Supplementary Fig. 1), 49 of which were hypomethylated, compared to 55 that were hypermethylated. 36 CpG sites were significantly differentially methylated (*P* adj < 0.05) at 7 y (whole blood, Table 2b, Supplementary Table 2 and Supplementary Fig. 2), 17 of which were hypomethylated, compared to 19 that were hypermethylated. 552 CpG sites were significantly differentially methylated (*P* adj < 0.05) at 15–17 y (whole blood, Table 2c, Supplementary Table 3 and Supplementary Fig. 3), 198 of which were hypomethylated, compared to 354 that were hypermethylated. The most significantly differentially methylated CpG sites with an associated gene at each timepoint were in the genes *TUBB2B* (0 y, *P* adj = 0.0160), *LZTS2* (7 y, *P* adj = 0.0004) and *WAC* (15–17 y, *P* adj = 0.00005). All EWAS were assessed for genomic inflation, with lambda values calculated as: 0 y, 0.768; 7 y, 1.301; 15–17 y, 0.794 (Supplementary Fig. 4a–c).

**Table 2:** The top 20 differentially methylated CpG sites in response to PCE at 0 y (2a, ALSPAC), 7 y (2b, ALSPAC), 15–17 y (2c, ALSPAC), and PCTE at ~27 y (2d, CHDS).

Rank	IlmnID	Gene Name	CHR	Beta difference	logFC	*P* value	adj. *P* val
**a**)							
1	cg22272277		7	0.0003	0.0046	1.42E−08	0.0067
2	cg23801012	*TUBB2B*	6	−0.0005	0.0113	6.94E−08	0.0160
3	cg18488855	*NOVA1*	14	0.0098	0.0067	2.23E−07	0.0160
4	cg23837191		18	−0.0030	0.0226	2.28E−07	0.0160
5	cg25533519		21	−0.0057	−0.0152	2.31E−07	0.0160
6	cg11818867	*OGFR*	20	−0.0010	0.0071	3.57E−07	0.0160
7	cg27551657	*TAF13*	1	0.0001	0.0085	3.65E−07	0.0160
8	cg17463149	*PKP1*	1	0.0063	0.0103	3.69E−07	0.0160
9	cg21201659	*SEC23IP*	10	0.0002	0.0029	4.23E−07	0.0160
10	cg09048530	*FZD10*	12	0.0015	0.0068	4.43E−07	0.0160
11	cg16109817	*FLJ37453*	1	−0.0027	0.0140	4.64E−07	0.0160
12	cg14528525	*C19orf48*	19	−0.0007	0.0114	5.31E−07	0.0160
13	cg14932794	*TOM1L1*	17	0.0003	0.0018	5.78E−07	0.0160
14	cg22256604	*STARD3*	17	0.0007	0.0081	6.00E−07	0.0160
15	cg08930904		17	−0.0083	−0.0120	6.02E−07	0.0160
16	cg01911440	*RPTOR*	17	−0.0115	−0.0175	6.05E−07	0.0160
17	cg19141861	*COQ5*	12	−0.0003	0.0039	6.11E−07	0.0160
18	cg04802236	*RPL23*	17	0.0002	0.0057	6.71E−07	0.0162
19	cg02742186	*CRYL1*	13	0.0004	0.0067	7.29E−07	0.0162
20	cg08479688	*TARBP1*	1	0.0000	0.0063	7.74E−07	0.0162
**b**)							
1	cg10170214	*LZTS2*	10	−0.0009	−0.0092	9.12E-10	0.0004
2	cg25208479		15	0.0017	0.0115	1.09E−07	0.0254
3	cg16028064	*BPTF*	17	−0.0002	0.0020	2.39E−07	0.0254
4	cg02850468	*NPSR1*	7	−0.0403	−0.0222	2.53E−07	0.0254
5	cg23992470	*GAK*	4	−0.0033	−0.0104	5.15E−07	0.0354
6	cg10872815	*PDE7B*	6	−0.0069	−0.0109	5.39E−07	0.0354
7	cg00731404	*MTIF3*	13	0.0000	0.0032	6.65E−07	0.0354
8	cg11681126	*ZNF32*	10	0.0006	0.0114	7.27E−07	0.0354
9	cg23938542	*CRIP2*	14	0.0007	0.0035	7.58E−07	0.0354
10	cg00646883	*COX18*	4	0.0019	0.0111	8.82E−07	0.0354
11	cg17721710	*SLC30A10*	1	0.0003	0.0024	9.32E−07	0.0354
12	cg14344315		15	0.0034	0.0027	1.08E−06	0.0354
13	cg23762037	*TUBGCP6*	22	0.0001	0.0016	1.18E−06	0.0354
14	cg19906737	*C8orf41*	8	−0.0001	0.0048	1.37E−06	0.0354
15	cg13770088	*PRRG4*	11	−0.0001	0.0021	1.53E−06	0.0354
16	cg18654873	*MAP3K7*	6	0.0002	0.0038	1.53E−06	0.0354
17	cg18262051	*MEPCE*	7	0.0046	0.0041	1.65E−06	0.0354
18	cg04010471		13	0.0001	0.0020	1.65E−06	0.0354
19	cg20514239	*RBP2*	3	−0.0174	−0.0245	1.67E−06	0.0354
20	cg18112005	*C14orf80*	14	0.0003	0.0043	1.68E−06	0.0354
**c**)							
1	cg13799287	*WAC*	10	0.0001	0.0063	2.20E-10	5.16E−05
2	cg20249919	*PCSK6*	15	0.0024	0.0089	2.84E-10	5.16E−05
3	cg14316565	*CAT*	11	0.0023	0.0077	3.28E-10	5.16E−05
4	cg14703784	*RASA3*	13	0.0002	−0.0101	6.50E-10	7.68E−05
5	cg05165940		2	0.0049	0.0220	8.29E-10	7.83E−05
6	cg01450600	*FAM160B1*	10	−0.0069	−0.0183	5.36E−09	0.0004
7	cg13692446		13	0.0009	0.0070	2.16E−08	0.0013
8	cg21108767	*PILRB*	7	−0.0010	0.0092	3.32E−08	0.0017
9	cg10888941		2	0.0048	0.0068	3.99E−08	0.0019
10	cg14283922	*ACBD3*	1	0.0017	0.0088	5.44E−08	0.0022
11	cg15994321	*ATF6*	1	0.0002	0.0033	5.73E−08	0.0022
12	cg03886242	*NFE2L3*	7	0.0005	0.0017	5.96E−08	0.0022
13	cg16528511	*RIPPLY2*	6	0.0032	0.0048	7.49E−08	0.0025
14	cg25559490	*WRNIP1*	6	0.0004	0.0066	9.74E−08	0.0029
15	cg10416994	*FBXO21*	12	−0.0012	0.0056	1.04E−07	0.0029
16	cg11443159	*IRGQ*	19	0.0061	0.0068	1.09E−07	0.0029
17	cg16174609	*R3HDML*	20	0.0005	−0.0145	1.10E−07	0.0029
18	cg23494140	*EDNRB*	13	0.0021	0.0068	1.27E−07	0.0032
19	cg24635468	*NT5E*	6	−0.0003	0.0020	1.36E−07	0.0032
20	cg07479092	*PLK2*	5	0.0037	0.0078	1.46E−07	0.0033
**d**)							
1	cg01483824	*GRIN2D*	19	−0.1303	−0.1404	1.48E-10	5.63E−05
2	cg00007036	*ZNF362*	1	−0.1265	−0.1352	2.79E-10	5.63E−05
3	cg24534173	*SPATA22*	17	−0.2623	−0.2776	2.87E-10	5.63E−05
4	cg16034787	*SLC9A3*	5	−0.3313	−0.3447	3.75E-10	5.63E−05
5	cg16326123	*HDAC4*	2	−0.1098	−0.1162	4.79E-10	5.63E−05
6	cg01311063		2	−0.1439	−0.1556	6.12E-10	5.63E−05
7	cg02144266	*C20orf135*	20	−0.1248	−0.1355	6.44E-10	5.63E−05
8	cg05174710	*RUNX1*	21	−0.2468	−0.2585	6.44E-10	5.63E−05
9	cg02698990*		6	−0.6245	−0.6284	3.40E−09	0.0003
10	cg19541688	*DMWD*	19	−0.1395	−0.1461	4.18E−09	0.0003
11	cg10086072*	*LPAR1*	9	−0.2381	−0.2064	1.56E−08	0.0009
12	cg16628641	*VASN*	16	−0.0697	−0.0784	1.62E−08	0.0009
13	cg01666550	*LRP5*	11	−0.3310	−0.3487	2.02E−08	0.0011
14	cg26306476	*PRR22*	19	−0.2331	−0.2449	2.23E−08	0.0011
15	cg16324015*	*SLC38A3*	3	−0.6517	−0.6592	2.88E−08	0.0013
16	cg12525596	*GOLGA3*	12	−0.6627	−0.6689	4.09E−08	0.0018
17	cg20227471	*ADCY3*	2	−0.4592	−0.4973	5.56E−08	0.0023
18	cg23367339	*ARHGAP23*	17	−0.5325	−0.5396	6.34E−08	0.0025
19	cg05262724	*PDZD2*	5	−0.4150	−0.4301	7.25E−08	0.0027
20	cg15159625	*MAN1A2*	1	−0.2121	−0.2092	1.34E−07	0.0047
21	cg13470557	*C20orf20*	20	−0.1508	−0.1654	1.53E−07	0.0051
22	cg18924102*	*SHANK2*	11	−0.6460	−0.6504	7.48E−07	0.0238
23	cg24641214*	*MOGAT3*	7	−0.6265	−0.6210	1.08E−06	0.0325
24	cg14080585	*TAF4*	20	−0.3175	−0.3323	1.11E−06	0.0325

*IlmnID* Illumina array probe ID, *CHR* chromosome number; beta difference, methylation difference between cases and controls, *logFC* log fold change of methylation difference. Empty cells are those which are not annotated to a gene or to a specific classification of CpG location. NB. 24 loci are presented in Table 2d instead of 20, as this was the final number of significantly differentially methylated probes in this analysis (supplement with the full list was not necessary). IlmnIDs in Table 2d marked with asterisks denote probes which are present on the 850 K array only.

### Overlap of CpG sites between 0 y, 7 y, and 15-17 y associated with prenatal cannabis exposure

**Fig. 1:**
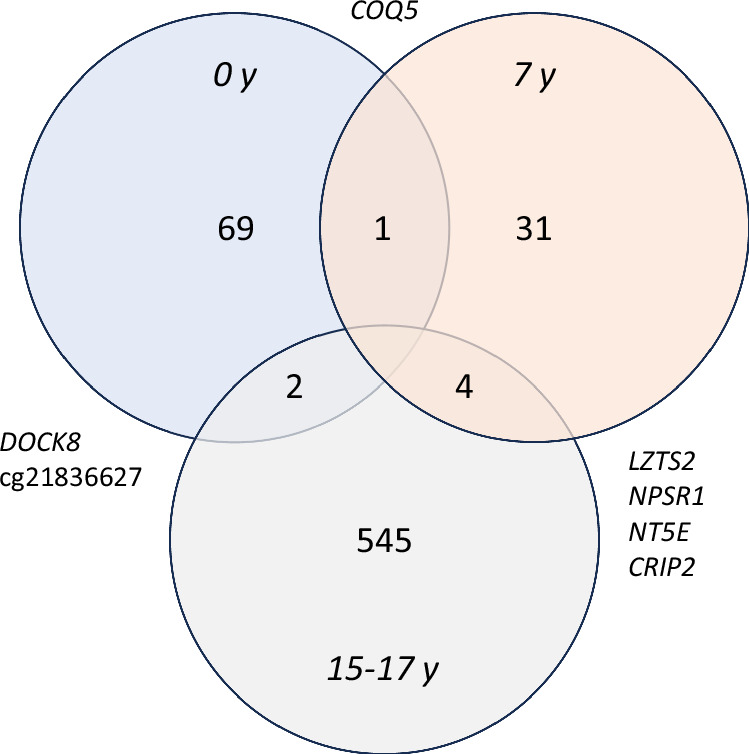
Significantly differentially methylated CpG sites in response to PCE that overlap between each ALSPAC timepoint analysed (0 y, 7 y and 15–17 y). The genes in which the CpG sites reside are listed external to the circles.

EWAS data was investigated for overlap of significantly differentially CpG methylated sites between all time points in the ALSPAC cohort (Fig. 1 and Table 3). Between 0 y and 7 y, one CpG site was shared (cg19141861 *COQ5*). A total of four CpG sites were shared between 7 y and 15-17 y (cg10170214 *LZTS2*, cg02850468 *NPSR1*, cg24635468 *NT5E*, and cg18576588 *CRIP2*) and a further two CpG sites shared between 15–17 y and 0 y (cg19201719 *DOCK8*, cg21836627).

**Table 3:** Significantly differentially methylated CpG sites in response to PCE that overlap between each ALSPAC timepoint analysed (0 y, 7 y and 15–17 y).

IlmnID	Gene Name	logFC 0 y	adj.*P*.Val 0 y	logFC 7 y	adj.*P*.Val 7 y	logFC 15-17 y	adj.*P*.Val 15-17 y
cg19141861	*COQ5*	0.0039	0.0160	0.0031	0.0457		
cg10170214	*LZTS2*			−0.0092	0.0004	−0.0072	0.0086
cg02850468	*NPRS1*			−0.0222	0.0254	−0.0174	0.0379
cg24635468	*NT5E*			0.0035	0.0362	0.0020	0.0032
cg18576588	*CRIP2*			0.0040	0.0427	0.0026	0.0045
cg19201719	*DOCK8*	0.0033	0.0267			0.0060	0.0116
cg21836627		0.0018	0.0282			0.0065	0.0102

*IllmID* Illumina array probe ID, *locFC* log fold change of methylation difference, *adj.p.val* adjusted *p* value of the methylation difference.

### Genome-wide DNA methylation analysis of prenatal cannabis and co-exposure with tobacco in the CHDS cohort at 27 y

EWAS was undertaken with the CHDS cohort. With Benjamini-Hochberg (BH) false discovery rate (FDR) correction for multiple testing, 24 CpG sites were significantly associated with either prenatal cannabis (PCE) and cannabis and tobacco (PCTE) exposure (whole blood, Table 2d and Supplementary Fig. 5). All differentially methylated CpG sites were hypomethylated. The most significantly differentially methylated CpG site was in the gene *GRIN2D* (*P* adj = 0.00006). The EWAS was assessed for genomic inflation, with lambda calculated as 1.714 (Supplementary Fig. 4d).

### Genome-wide DNA methylation of prenatal tobacco only exposure (PTE) in the CHDS cohort at 27 y

To assess the effect of PCE only on DNA methylation, vs. any confounding factors that may be present in co-exposure with tobacco, we performed an additional EWAS on PCE and PCTE individuals, compared to non-exposed controls, with the analysis instead based on prenatal tobacco exposure (PTE), that is, corrected for prenatal cannabis exposure and adult cannabis and tobacco use. A total of two CpG sites both within the gene *FRMD4A* were significantly differentially methylated (*P* adj < 0.05), with one additional differentially methylated CpG site in the same not reaching the FDR cut-off for genome-wide significance (Supplementary Table 4 and Supplementary Fig. 6). The EWAS was assessed for genomic inflation, with lambda calculated as 0.838 (Supplementary Fig. 4e)

### Overlap of significantly differentially methylated CpG sites between ALSPAC and CHDS

Significant differential methylation at one CpG site (cg01666550) within the gene body of *LRP5* (chr11:68181086) was shared between the ALSPAC cohort at age 15–17 y (rank 448 of 552, log fold change −0.0066, *P* adj = 0.0255) and CHDS cohort at age 27 y (rank 13 of 24, log fold change −0.3487, *P* adj = 0.0011) in whole blood. Five out of the 24 most significantly differentially methylated CpG sites in the CHDS cohort were probes that were only present on the 850 K array, and were absent from the 450 K array (probes ranked 9, 11, 15, 22 and 22, Table 2d).

### Pathway analyses

At 0 y, 7 y, 15 y, and 27 y, gene set enrichment indicated that differential DNA methylation was enriched at loci that are annotated to multiple gene ontology pathways associated with neurodevelopment, neurotransmission, regulation of neural pathways, and neuronal structure, as well as molecular pathways that have associations with neurodevelopmental diseases and disorders (Table 4).

**Table 4:** Gene ontology enrichment analysis for GO terms in response to PCE (ALSPAC) and PCTE (CHDS).

GO	Description	type	DMG	background	*p*	FDR
**ALSPAC PCE 0 y**						
GO:0048665	neuron fate specification	BP	18	34	9.44E−05	1
GO:0048663	neuron fate commitment	BP	26	65	1.34E−03	1
GO:0030594	neurotransmitter receptor activity	MF	31	115	1.48E−03	1
GO:0001708	cell fate specification	BP	34	99	1.76E−03	1
GO:0099566	regulation of postsynaptic cytosolic calcium ion concentration	BP	8	12	1.84E−03	1
GO:0004505	phenylalanine 4-monooxygenase activity	MF	3	3	2.57E−03	1
GO:0061304	retinal blood vessel morphogenesis	BP	5	6	4.14E−03	1
GO:0016616	oxidoreductase activity, acting on the CH-OH group of donors, NAD or NADP as acceptor	MF	28	118	4.54E−03	1
GO:0004970	ionotropic glutamate receptor activity	MF	10	19	4.60E−03	1
GO:0007379	segment specification	BP	8	17	4.95E−03	1
**ALSPAC PCE 7** **y**						
GO:0051899	membrane depolarisation	BP	31	91	0.0003	1
GO:0017156	calcium ion regulated exocytosis	BP	46	152	0.0007	1
GO:0043196	varicosity	CC	6	8	0.0010	1
GO:0016079	synaptic vesicle exocytosis	BP	38	118	0.0010	1
GO:0005854	nascent polypeptide-associated complex	CC	4	5	0.0010	1
GO:0098797	plasma membrane protein complex	CC	134	563	0.0012	1
GO:0003714	transcription corepressor activity	MF	62	233	0.0014	1
GO:0032892	positive regulation of organic acid transport	BP	12	33	0.0015	1
GO:0046717	acid secretion	BP	35	124	0.0015	1
GO:0098742	cell-cell adhesion via plasma-membrane adhesion molecules	BP	69	271	0.0016	1
**ALSPAC PCE 15-17** **y**						
GO:1901533	negative regulation of hematopoietic progenitor cell differentiation	BP	6	8	0.0009	1
GO:0050905	neuromuscular process	BP	36	107	0.0012	1
GO:0032885	regulation of polysaccharide biosynthetic process	BP	17	37	0.0016	1
GO:0035254	glutamate receptor binding	MF	20	46	0.0024	1
GO:0032881	regulation of polysaccharide metabolic process	BP	18	42	0.0025	1
GO:0005979	regulation of glycogen biosynthetic process	BP	14	29	0.0025	1
GO:0010962	regulation of glucan biosynthetic process	BP	14	29	0.0025	1
GO:0031646	positive regulation of neurological system process	BP	25	63	0.0028	1
GO:0060364	frontal suture morphogenesis	BP	4	4	0.0032	1
GO:0006750	glutathione biosynthetic process	BP	9	17	0.0035	1
**CHDS PCTE 27** **y**						
GO:0022038	corpus callosum development	BP	10	16	0.0006	1
GO:0035927	RNA import into mitochondrion	BP	4	5	0.0009	1
GO:0010842	retina layer formation	BP	12	22	0.0013	1
GO:0060563	neuroepithelial cell differentiation	BP	21	53	0.0016	1
GO:1900086	positive regulation of peptidyl-tyrosine autophosphorylation	BP	4	4	0.0016	1
GO:1902285	semaphorin-plexin signalling pathway involved in neuron projection guidance	BP	9	13	0.0018	1
GO:0045747	positive regulation of Notch signalling pathway	BP	21	52	0.0018	1
GO:0021532	neural tube patterning	BP	17	40	0.0018	1
GO:0061478	response to platelet aggregation inhibitor	BP	8	13	0.0029	1
GO:0072300	positive regulation of metanephric glomerulus development	BP	4	5	0.0033	1

The top 10 biological processes that are significantly enriched in differential methylation data at 0 y, 7 y, and 15–17 y (ALSPAC) and at 27 y (CHDS) are presented. The type of gene ontology term in the table is listed as BP (biological process), MF (molecular function), or CC (cellular component). Each biological process is listed with its gene set identifier from the Gene Ontology database. DMG, differentially methylated gene; background, the number of genes in the GO term against which the presence of DMGs are assessed; P, *p*-value of the relationship between DMGs and background; FDR, FDR-corrected *p* value of this relationship.

## Discussion

To date, limited research has explored molecular changes in offspring associated with prenatal cannabis exposure (PCE). In this study, we performed epigenome-wide association studies of PCE, and prenatal cannabis and tobacco exposure (PCTE), in two independent cohorts at four time points; 0 y, 7 y, 15-17 y, and ~27 y. Our findings show that both PCE and PCTE are associated with genome-wide significant DNA methylation differences at all time points, in both cohorts, at genes, and in pathways associated with neurodevelopment. These results provide molecular evidence in support of the epidemiological associations between PCE and impacts on neurodevelopment. Further, they provide justification for larger studies in this area and support further conversations around the need for guidelines to discuss a reduction in prenatal cannabis exposure.

### Prenatal cannabis exposure and differential DNA methylation in the ALSPAC cohort

To assess the impact of PCE on DNA methylation we undertook EWAS at three time points, including birth (0 y), early childhood (7 y), and adolescence (15–17 y), in the ALSPAC cohort. The top PCE-associated differentially methylated CpG site at 0 y was in the gene *TUBB2B*. This gene is associated with malformations of cortical development [67], and mutations in *TUBB2B* are associated with developmental delays [68]. The top CpG site displaying differential methylation at 7 y was in the gene *LZTS2*. Differential methylation of *LZTS2* is associated with cross-generational effects of THC exposure in rat nucleus accumbens [48]. Further, *LZTS2* is associated with major depressive disorder [69]. Lastly, the top differentially methylated CpG site at age 15 y was in the gene *WAC*, which has been linked to severe intellectual disability [70].

We detected varying abundances of differentially methylated loci between early childhood and adolescence in these data, which was not unexpected; it is well established that genome-wide levels of DNA methylation change over time and that stochastic methylation changes occur during the ageing process [71], and this pattern reflected in these data. However, the varying methylation abundance with age also means that identifying consistent changes between childhood, adolescence, and adulthood, where differential methylation is expected to be more prominent [71], based on each individual’s environmental exposures, and related to exposures during development, is challenging. Despite this challenge, in these data, we identified shared differential DNA methylation at individual CpG sites between different time points examined in the ALSPAC cohort. Specifically, between individuals at 7 y and 15 y, differential DNA methylation was shared at 4 CpG sites. These sites were in or near the genes *LZTS2* (discussed above)*, NPSR1, NT5E*, and *CRIP2*. *NPSR1* is associated with educational attainment [72] and amyloid beta measurement [73]. *NT5E* may have a role in excitatory neurotransmission [74]. *CRIP2* currently has no known roles in neurodevelopment or neurodevelopmental disorders, however, is associated with heart development [75, 76] which is intriguing as growing evidence suggests that PCE is associated with impacts on heart development [77], and further, adult cannabis use is known to impact DNA methylation in biological pathways associated with cardiomyopathy [78]. Next, we detected significant differential DNA methylation shared between individuals at 0 y and 15 y at 2 CpG sites, in response to PCE. These sites were in the gene *DOCK8*, and an un-annotated CpG, with probe ID cg21836627. *DOCK8* is associated with anxiety [79], opioid dependence [80], and autism spectrum disorder (ASD) [81]. The probe cg21836627 is not within or directly associated with a gene (chr6:30,069,797). However, this CpG site is 877 bp upstream of *TRIM31* (chr6:30,070,674-30,080,867), which is associated with cognitive performance/intelligence [82, 83], memory [83], unipolar depression [83, 84], ASD and schizophrenia [85], all of which are epidemiologically associated with PCE. Differential methylation at one site was shared between 0 y and 7 y, and this site was in the gene *COQ5*. *COQ5* is a methyltransferase enzyme [86] that is located in the mitochondria. *COQ5* has associations with educational attainment [87], global developmental delay [88], and intellectual disability [89]. Taken together, these findings imply an impact of PCE on DNA methylation at genes with roles in neurodevelopment, across childhood and adolescence.

Given these data indicate an impact on genes involved in neurodevelopment, we undertook gene ontology enrichment analyses to determine whether this pattern was supported at the molecular pathway level. Our data indicated that differential methylation is significantly enriched at pathways associated with neurodevelopment, but also neurotransmission, regulation of neural pathways, and neuronal structure, at 0 y, 7 y, and 15-17 y. The signature of enrichment, in response to prenatal exposure, supports the principle that many neurodevelopmental disorders and diseases have developmental origins, and is also supportive of the epidemiological associations between PCE and neurodevelopment. For example, PCE is associated with consequences for neurodevelopment [25], potentially via disruption of the developing central nervous system by cannabinoid exposure [36, 90], which could impact neuron development and growth [91]. This potential impact on the developing nervous system may be linked to the association between PCE and an increase in psychosis and psychopathology in middle childhood [24, 92], and the greater risk of psychiatric disorders into adolescence [93], including affective symptoms and ADHD [94]. Further, PCE is associated with impacts on memory and behaviour in children and adolescents, and this impact persists into adulthood [26]. Thus, along with the frequent and wide-ranging reviews on the association between PCE and neurodevelopment and behaviour across the lifespan [39, 95], and recent evidence of an impact of PCE on DNA methylation at genes involved in autism in animal models [49], taken together, these data support a role for DNA methylation in the biological response to PCE.

### Prenatal cannabis and tobacco exposure and differential DNA methylation in the CHDS cohort

In the CHDS cohort, we detected 24 significantly differentially methylated CpG sites, in individuals exposed to cannabis and tobacco during development, compared to controls. The most significantly differentially methylated sites were in the genes: *GRIN2D*, which is a glutamate receptor gene that functions in synaptic transmission and is involved in long-term potentiation/learning and memory [96, 97]; *ZNF362*, which has associations with individual schizophrenia symptom severity [98]; *SPATA2*, which is involved in fertilisation, but also intelligence [99], attention deficit hyperactivity disorder (ADHD) and ASD [100]; *SLC9A3*, an Na^+^/H^+^ exchanger, classes of which have an emerging role in developmental brain disorders [101], and finally; *HDAC4*, which has been associated with ADHD, uni- and bipolar depression, ASD and schizophrenia [102], educational attainment [87], as well as a prominent role in Brachydactyly Mental Retardation Syndrome, symptoms of which commonly include cognitive delay [103, 104] and autistic-like behaviours [105].

Gene ontology enrichment analysis for CHDS confirmed that differential methylation of genes involved in neurodevelopment and neurodevelopmental disorders were supported at the pathway level. We identified enrichment for the terms neuroepithelial cell differentiation, neural tube patterning, and neuron projection guidance, indicating enrichment for genes associated with neurogenesis and neural connections. Additionally, differential methylation is enriched at genes associated with corpus collosum development. The corpus collosum is consistently found to be reduced in size in patients with ASD [106] and schizophrenia [107], and is thought to have a role in neurodevelopmental disorders such as ASD and ADHD [108]. Further enriched is the GO term ‘positive regulation of peptidyl-tyrosine autophosphorylation’. This GO term is enriched in studies of ASD [109] and also neurodevelopmental disorders/neuromotor development [110]. Finally, loci within the Notch signalling pathway are enriched in these data. Notch is integral in nervous system development and influences plasticity and learning in the adult central nervous system [111]. Altered Notch signalling has been associated with ASD [112], schizophrenia and bipolar disorder [113]. Thus, taken together, pathway enrichment analyses support the finding that the most significantly differentially methylated loci function in neurodevelopment and neurodevelopmental disorders, as well as provide molecular evidence in support the epidemiological associations of a link between PCE and these disorders.

To understand whether an analysis of cannabis and tobacco exposure (PCTE), in combination with cannabis-only exposure (PCE), was skewing these data in favour of tobacco exposure-driven differential methylation, we assessed the same DNA methylation data and asked the extent of differential DNA methylation in response to prenatal tobacco exposure alone (PTE), corrected for PCE and adult cannabis and tobacco use. This EWAS detected genome-wide significant DNA methylation at two CpG sites, both within the gene Ferm domain containing 4 A (*FRMDA4)*. Previous studies have found *FRMDA4* to be differentially methylated in response to prenatal tobacco exposure, with the strongest effects observed in sustained prenatal tobacco smoking [114]. Thus, while we were unable to control for PTE in our CHDS analysis, our EWAS observations of both PCTE and PTE support a distinct pattern of differential DNA methylation that is driven by prenatal exposure to cannabis, in adult offspring.

### Overlap in differentially methylated CpG sites between the ALSPAC and CHDS cohorts

One CpG site in the gene *LRP5* was differentially methylated in CHDS adults and in the eldest time point (15–17 y) in the ALSPAC cohort. *LRP5* has not yet been associated with PCE, and therefore may represent a novel target for PCE. The *LRP5* gene codes for low-density lipoprotein receptor-related protein 5, and differential methylation of CpG sites within this gene have previously been associated with passive smoking exposure [115], and implicated in the proposed link between cigarette smoking habit and metabolic syndrome [116]. Moreover, it has associations with individual educational attainment [72] and mathematical ability [87], which supports further investigation of this gene for its role in the molecular link between PCE and offspring neurodevelopment.

### Strengths and challenges associated with cross-cohort DNA methylation analyses

It is important to acknowledge the challenges associated with our study. Firstly, while our study identifies DNA methylation changes at loci and pathways that support the epidemiological associations of PCE, at all time points, which reflects the strength of our hypothesis, our study consists of a relatively small number of individuals with prenatal exposure to cannabis, and as such, we must emphasise the need for replication in a more highly powered study cohort. Secondly, using independent data from multiple cohorts, as we have done here, can pose additional challenges, including those associated with attempting to validate differential methylation between samples at different ages, and differences between workflows, including normalisation and quality control steps, where raw data is not available. Further, combining and validating across age points and independent datasets is even more challenging when one considers confounding factors such as tobacco, alcohol, and other drug use; these common confounders lead to heterogeneity in results cross-cohort, and along with sociodemographic factors, makes uniformity across studies hard to achieve [117]. This is pertinent when we consider the genomic inflation values for the 15–17 y time point; the lambda value here indicates a slight inflation. We predict that this is due to the available variables from the ALSPAC study; clinical data was only collected at 7 y, meaning we were unable to correct for personal tobacco smoking status, which is relevant at this time point. We suggest that this might be a possible explanation for the inflation value and the increase in the number of differentially methylated probes observed at this time point, however, without access to more clinical data, we are unable to correct for this in our EWAS model. Therefore, while identifying independent cohorts that reliably and consistently measure all possible confounding variables is not possible or feasible, it nevertheless remains a limitation of studies such as this. We also acknowledge the limitations that surround the necessary use of DNA from blood samples in this study, which we are aware may or may not reflect changes in the brain. Furthermore, due to the aforementioned limitations around study size, it was necessary to analyse PCE in combination with tobacco (PCTE) for one cohort, and we acknowledge that, despite controlling for tobacco exposure, we cannot ignore this as a potential confounder in these data. Nevertheless, while both cannabis and tobacco can result in different changes to the methylome, and while there is a large proportion of PCE individuals who have co-exposure with tobacco, we demonstrated that many significant CpG sites are unique to prenatal cannabis exposure in the CHDS. Moreover, we demonstrate that each dataset, whether PCE or PCTE, is enriched for similar/shared biological pathways, giving us confidence that our data are supportive of a biological role for DNA methylation in the association between PCE and neurodevelopment, and that further investigation in larger cohorts is required.

## Conclusion

These data provide hypothesis-building insights into molecular alterations associated with prenatal cannabis exposure. The association between DNA methylation and genes involved in neurodevelopment and neurodevelopmental disorders in the present study may suggest a role for DNA methylation in the link between PCE and neurodevelopmental outcomes in exposed children, as well as provide a suite of reference genes for future investigations into the molecular mechanism of the impact of PCE on individuals. While both of our two independent cohorts contain modest sample numbers of exposed individuals, finding suitable cohorts with defined and comprehensive data on PCE is a difficult task, and the biological relevance of these data to the phenotypes being studied provides justification that PCE should be investigated strategically and specifically, in much larger sample sizes. Given the increasing prevalence of PCE, further research is required to identify the underlying mechanisms that may underlie the effects of PCE on neurodevelopment, in order to start to establish whether PCE is causative for neurodevelopmental impacts. Moreover, these data will help to fill current gaps in our knowledge around how drug exposures during development may lead to lifelong molecular disturbances that predispose an individual to poor neurocognitive outcomes in early childhood, adolescence, and beyond [39].

## Supplementary information

Supplementary Information

Supplementary Table 1

Supplementary Table 2

Supplementary Table 3

Supplementary Table 4

Supplementary Fig. 1

Supplementary Fig. 2

Supplementary Fig. 3

Supplementary Fig. 4

Supplementary Fig. 5

Supplementary Fig. 6

## Data Availability

CHDS – Data available on request.

## Code availability

The computer codes used to generate these results may be requested via the corresponding author.
