# Supplementary material for: Prenatal cannabis exposure is associated with alterations in offspring DNA methylation at genes involved in neurodevelopment, across the life course
Source: Mol Psychiatry. 2024 Sep 14;30(4):1418–29. doi: 10.1038/s41380-024-02752-w (PMC11919715; doi:10.1038/s41380-024-02752-w)
Supplement: Supplementary file 1 — Supplementary Information [file 41380_2024_2752_MOESM1_ESM.docx]

**Supplementary Information**

**Supplementary Figures**

**Supplementary Figure 1**

An epigenome-wide association study, presented as a Manhattan plot, of cases (individuals exposed to cannabis only (N = 10) or cannabis with tobacco (N = 20) vs. controls (N = 654) in the ALSPAC cohort, at 0 y. Data is corrected for prenatal tobacco exposure status, sex and cell type proportions. The dotted line denotes the smallest -log10(p-value) that is below the FDR correction threshold of 0.05.

**Supplementary Figure 2**

An epigenome-wide association study, presented as a Manhattan plot, of cases (individuals exposed to cannabis only (N = 9) or cannabis with tobacco (N = 22) vs. controls (N = 700) in the ALSPAC cohort, at 7 y. Data is corrected for prenatal tobacco exposure status, sex and cell type proportions. The dotted line denotes the smallest -log10(p-value) that is below the FDR correction threshold of 0.05.

**Supplementary Figure 3**

An epigenome-wide association study, presented as a Manhattan plot, of cases (individuals exposed to cannabis only (N = 11) or cannabis with tobacco (N = 21) vs. controls (N = 701) in the ALSPAC cohort, at 15-17 y. Data is corrected for prenatal tobacco exposure status, sex and cell type proportions. The dotted line denotes the smallest -log10(p-value) that is below the FDR correction threshold of 0.05.

**Supplementary Figure 4**

Q-Q plots of genomic inflation for PCE at 0 y (a), 7 y (b) and 15-17 y (c), PCTE at ~27 y (d) and PTE at ~27 y (e).

**Supplementary Figure 5**

An epigenome-wide association study, presented as a Manhattan plot, of cases (individuals exposed to cannabis only (N = 4) or cannabis with tobacco (N = 9)) vs. controls (N = 85) in the CHDS cohort. Data is corrected for adult tobacco smoking status, sex and cell type proportion, adult cannabis use. The dotted line denotes the smallest -log10(p-value) that is below the FDR correction threshold of 0.05.

**Supplementary Figure 6**

An epigenome-wide association study of differential methylation in response to prenatal tobacco exposure (PTE, N = 55), corrected for prenatal cannabis exposure (N = 13), adult smoking status, sex and cell type proportion in the CHDS cohort. Two CpG sites in one gene (*FRMD4A*) are significantly differentially methylated in response to PTE at the genome-wide level. The dotted line denotes the smallest -log10(p-value) that is below the FDR correction threshold of 0.05.

**Supplementary Tables**

**Supplementary Table 1**

All significantly differentially methylated CpG sites in response to PCE at 0 y (ALSPAC). IlmnID, Illumina array probe ID; CHR, chromosome number; beta difference, methylation difference between cases and controls; logFC, log fold change of methylation difference. Empty cells are those which are not annotated to a gene or to a specific classification of CpG location.

**Supplementary Table 2**

All significantly differentially methylated CpG sites in response to PCE at 7 y (ALSPAC). IlmnID, Illumina array probe ID; CHR, chromosome number; beta difference, methylation difference between cases and controls; logFC, log fold change of methylation difference. Empty cells are those which are not annotated to a gene or to a specific classification of CpG location.

**Supplementary Table 3**

All significantly differentially methylated CpG sites in response to PCE at 15-17 y (ALSPAC). IlmnID, Illumina array probe ID; CHR, chromosome number; beta difference, methylation difference between cases and controls; logFC, log fold change of methylation difference. Empty cells are those which are not annotated to a gene or to a specific classification of CpG location.

**Supplementary Table 4**

The top 20 differentially methylated CpG sites in response to PTE at ~27 y (CHDS). IlmnID, Illumina array probe ID; CHR, chromosome number; beta difference, methylation difference between cases and controls; logFC, log fold change of methylation difference. Empty cells are those which are not annotated to a gene or to a specific classification of CpG location.
