# Supplementary material for: Prenatal cannabis exposure is associated with alterations in offspring DNA methylation at genes involved in neurodevelopment, across the life course
Source: Mol Psychiatry. 2024 Sep 14;30(4):1418–29. doi: 10.1038/s41380-024-02752-w (PMC11919715; doi:10.1038/s41380-024-02752-w)
Supplement: Supplementary file 2 — Supplementary Table 1 [file 41380_2024_2752_MOESM2_ESM.docx]

**Supplementary Table 1**

All significantly differentially methylated CpG sites in response to PCE at 0 y (ALSPAC).

| **Rank** | **IlmnID** | **Gene Name** | **CHR** | **Beta difference** | **logFC** | **P.Value** | **adj.P.Val** |
| --- | --- | --- | --- | --- | --- | --- | --- |
| 1 | cg22272277 |  | 7 | 0.0003 | 0.0046 | 1.42E-08 | 0.0067 |
| 2 | cg23801012 | *TUBB2B* | 6 | -0.0005 | 0.0113 | 6.94E-08 | 0.0160 |
| 3 | cg18488855 | *NOVA1* | 14 | 0.0098 | 0.0067 | 2.23E-07 | 0.0160 |
| 4 | cg23837191 |  | 18 | -0.0030 | 0.0226 | 2.28E-07 | 0.0160 |
| 5 | cg25533519 |  | 21 | -0.0057 | -0.0152 | 2.31E-07 | 0.0160 |
| 6 | cg11818867 | *OGFR* | 20 | -0.0010 | 0.0071 | 3.57E-07 | 0.0160 |
| 7 | cg27551657 | *TAF13* | 1 | 0.0001 | 0.0085 | 3.65E-07 | 0.0160 |
| 8 | cg17463149 | *PKP1* | 1 | 0.0063 | 0.0103 | 3.69E-07 | 0.0160 |
| 9 | cg21201659 | *SEC23IP* | 10 | 0.0002 | 0.0029 | 4.23E-07 | 0.0160 |
| 10 | cg09048530 | *FZD10* | 12 | 0.0015 | 0.0068 | 4.43E-07 | 0.0160 |
| 11 | cg16109817 | *FLJ37453* | 1 | -0.0027 | 0.0140 | 4.64E-07 | 0.0160 |
| 12 | cg14528525 | *C19orf48* | 19 | -0.0007 | 0.0114 | 5.31E-07 | 0.0160 |
| 13 | cg14932794 | *TOM1L1* | 17 | 0.0003 | 0.0018 | 5.78E-07 | 0.0160 |
| 14 | cg22256604 | *STARD3* | 17 | 0.0007 | 0.0081 | 6.00E-07 | 0.0160 |
| 15 | cg08930904 |  | 17 | -0.0083 | -0.0120 | 6.02E-07 | 0.0160 |
| 16 | cg01911440 | *RPTOR* | 17 | -0.0115 | -0.0175 | 6.05E-07 | 0.0160 |
| 17 | cg19141861 | *COQ5* | 12 | -0.0003 | 0.0039 | 6.11E-07 | 0.0160 |
| 18 | cg04802236 | *RPL23* | 17 | 0.0002 | 0.0057 | 6.71E-07 | 0.0162 |
| 19 | cg02742186 | *CRYL1* | 13 | 0.0004 | 0.0067 | 7.29E-07 | 0.0162 |
| 20 | cg08479688 | *TARBP1* | 1 | 0.0000 | 0.0063 | 7.74E-07 | 0.0162 |
| 21 | cg09743140 | *WDR51B* | 12 | 0.0001 | 0.0062 | 8.20E-07 | 0.0162 |
| 22 | cg05045329 | *CNNM4* | 2 | -0.0004 | 0.0100 | 8.52E-07 | 0.0162 |
| 23 | cg17695351 | *HADHB* | 2 | 0.0003 | 0.0122 | 8.58E-07 | 0.0162 |
| 24 | cg03271965 | *TMEM216* | 11 | 0.0007 | 0.0038 | 8.58E-07 | 0.0162 |
| 25 | cg12069073 | *KDM2A* | 11 | 0.0001 | 0.0025 | 1.00E-06 | 0.0169 |
| 26 | cg10177056 | *RASGRP3* | 2 | -0.0087 | -0.0086 | 1.00E-06 | 0.0169 |
| 27 | cg24361256 | *SELO* | 22 | -0.0271 | -0.0196 | 1.05E-06 | 0.0171 |
| 28 | cg14350176 |  | 9 | -0.0001 | 0.0073 | 1.34E-06 | 0.0210 |
| 29 | cg04145937 |  | 1 | -0.0547 | -0.0188 | 1.43E-06 | 0.0218 |
| 30 | cg16755393 | *MAP4* | 3 | -0.0009 | 0.0114 | 1.49E-06 | 0.0219 |
| 31 | cg22578433 | *CCND3* | 6 | -0.0293 | -0.0204 | 1.58E-06 | 0.0219 |
| 32 | cg02384857 | *HOXA7* | 7 | 0.0060 | 0.0071 | 1.59E-06 | 0.0219 |
| 33 | cg07448606 | *PCDHGA4* | 5 | 0.0018 | 0.0053 | 1.66E-06 | 0.0219 |
| 34 | cg18504015 |  | 6 | 0.0000 | 0.0019 | 1.67E-06 | 0.0219 |
| 35 | cg00601648 | *ADCY8* | 8 | 0.0003 | -0.0116 | 1.79E-06 | 0.0226 |
| 36 | cg17124278 | *BAT4* | 6 | 0.0001 | 0.0066 | 1.82E-06 | 0.0226 |
| 37 | cg27081243 | *CUTA* | 6 | 0.0002 | 0.0050 | 1.89E-06 | 0.0229 |
| 38 | cg09366969 | *PDE4DIP* | 1 | 0.0144 | 0.0276 | 1.95E-06 | 0.0229 |
| 39 | cg25765619 | *MAFB* | 20 | 0.0025 | 0.0090 | 2.04E-06 | 0.0229 |
| 40 | cg01544903 |  | 16 | 0.0007 | 0.0049 | 2.08E-06 | 0.0229 |
| 41 | cg09668564 | *ABHD5* | 3 | 0.0110 | 0.0061 | 2.15E-06 | 0.0229 |
| 42 | cg04785675 |  | 1 | -0.0008 | 0.0031 | 2.19E-06 | 0.0229 |
| 43 | cg05873285 | *AIM1* | 6 | 0.0003 | 0.0034 | 2.20E-06 | 0.0229 |
| 44 | cg15645634 | *NR3C1* | 5 | 0.0001 | 0.0027 | 2.27E-06 | 0.0229 |
| 45 | cg15283373 | *DTX3* | 12 | -0.0006 | 0.0105 | 2.30E-06 | 0.0229 |
| 46 | cg13013671 | *CCDC112* | 5 | 0.0000 | 0.0115 | 2.57E-06 | 0.0248 |
| 47 | cg26678970 |  | 13 | 0.0023 | 0.0099 | 2.64E-06 | 0.0248 |
| 48 | cg26220033 | *SNX24* | 5 | -0.0007 | 0.0041 | 2.68E-06 | 0.0248 |
| 49 | cg13855924 | *KCNK17* | 6 | 0.0001 | 0.0031 | 2.75E-06 | 0.0249 |
| 50 | cg16948199 | *DHRS12* | 13 | -0.0003 | 0.0050 | 2.79E-06 | 0.0249 |
| 51 | cg17296482 | *CCND2* | 12 | -0.0004 | 0.0041 | 3.07E-06 | 0.0262 |
| 52 | cg00961326 | *MMP15* | 16 | -0.0012 | 0.0121 | 3.09E-06 | 0.0262 |
| 53 | cg03973167 | *IRF2* | 4 | -0.0001 | 0.0082 | 3.14E-06 | 0.0262 |
| 54 | cg03054162 | *PCYOX1* | 2 | -0.0011 | 0.0112 | 3.17E-06 | 0.0262 |
| 55 | cg04622176 | *SEC23IP* | 10 | 0.0003 | 0.0021 | 3.33E-06 | 0.0267 |
| 56 | cg19201719 | *DOCK8* | 9 | 0.0000 | 0.0033 | 3.36E-06 | 0.0267 |
| 57 | cg00734993 | *MTCH1* | 6 | 0.0016 | 0.0046 | 3.39E-06 | 0.0267 |
| 58 | cg05615019 |  | 7 | 0.0078 | 0.0054 | 3.60E-06 | 0.0275 |
| 59 | cg24076774 | *SRBD1* | 2 | -0.0016 | 0.0088 | 3.66E-06 | 0.0275 |
| 60 | cg10715223 | *SNX31* | 8 | -0.0003 | 0.0025 | 3.67E-06 | 0.0275 |
| 61 | cg27434863 | *RUNDC2A* | 16 | -0.0149 | -0.0113 | 3.75E-06 | 0.0277 |
| 62 | cg21836627 |  | 6 | 0.0004 | 0.0018 | 3.93E-06 | 0.0282 |
| 63 | cg01643123 | *PPM1H* | 12 | 0.0050 | 0.0176 | 3.95E-06 | 0.0282 |
| 64 | cg06463097 | *FASN* | 17 | -0.0021 | -0.0059 | 4.00E-06 | 0.0282 |
| 65 | cg15276500 | *ADRM1* | 20 | 0.0040 | 0.0088 | 4.27E-06 | 0.0296 |
| 66 | cg13272701 | *MAL2* | 8 | 0.0010 | 0.0078 | 4.32E-06 | 0.0296 |
| 67 | cg14083397 | *RBCK1* | 20 | -0.0014 | 0.0225 | 4.49E-06 | 0.0303 |
| 68 | cg23319285 |  | 10 | -0.0068 | 0.0291 | 4.80E-06 | 0.0314 |
| 69 | cg17773349 | *ATCAY* | 19 | 0.0020 | 0.0053 | 4.84E-06 | 0.0314 |
| 70 | cg17937102 | *ALDH9A1* | 1 | -0.0003 | 0.0120 | 4.86E-06 | 0.0314 |
| 71 | cg22876160 | *CYTH2* | 19 | 0.0007 | 0.0119 | 4.95E-06 | 0.0316 |
| 72 | cg22216738 |  | 19 | 0.0001 | 0.0029 | 5.35E-06 | 0.0333 |
| 73 | cg23419897 | *ATXN7L1* | 7 | 0.0016 | -0.0103 | 5.66E-06 | 0.0347 |
| 74 | cg13124263 | *C6orf57* | 6 | -0.0005 | 0.0032 | 5.80E-06 | 0.0351 |
| 75 | cg01450204 | *GALNTL4* | 11 | 0.0017 | 0.0101 | 6.02E-06 | 0.0351 |
| 76 | cg27550982 | *STXBP2* | 19 | -0.0001 | 0.0016 | 6.05E-06 | 0.0351 |
| 77 | cg14291622 | *PLIN2* | 9 | 0.0008 | 0.0009 | 6.08E-06 | 0.0351 |
| 78 | cg12601118 | *GAS7* | 17 | 0.0011 | -0.0055 | 6.10E-06 | 0.0351 |
| 79 | cg12518360 | *SLC36A4* | 11 | 0.0053 | 0.0070 | 6.23E-06 | 0.0354 |
| 80 | cg20303850 | *SLC25A3* | 12 | 0.0032 | 0.0045 | 6.51E-06 | 0.0366 |
| 81 | cg09667226 | *KLF5* | 13 | -0.0004 | 0.0065 | 6.85E-06 | 0.0381 |
| 82 | cg24205332 | *ADAMTS8* | 11 | -0.0001 | 0.0105 | 7.40E-06 | 0.0398 |
| 83 | cg10663055 | *NT5E* | 6 | 0.0017 | 0.0072 | 7.42E-06 | 0.0398 |
| 84 | cg12210286 | *CNTD2* | 19 | 0.0000 | 0.0021 | 7.51E-06 | 0.0398 |
| 85 | cg01408342 | *TMEM14A* | 6 | -0.0004 | 0.0025 | 7.52E-06 | 0.0398 |
| 86 | cg11788234 |  | 13 | 0.0001 | 0.0040 | 7.59E-06 | 0.0398 |
| 87 | cg04735123 | *MYL2* | 12 | -0.0002 | -0.0069 | 8.16E-06 | 0.0416 |
| 88 | cg11332236 | *RHCG* | 15 | 0.0001 | 0.0025 | 8.32E-06 | 0.0416 |
| 89 | cg01154849 | *NCRNA00188* | 17 | -0.0025 | 0.0099 | 8.33E-06 | 0.0416 |
| 90 | cg07862977 | *FGFR1* | 8 | -0.0009 | -0.0024 | 8.33E-06 | 0.0416 |
| 91 | cg20870360 |  | 2 | -0.0186 | -0.0122 | 8.37E-06 | 0.0416 |
| 92 | cg10143067 | *VAX1* | 10 | 0.0053 | 0.0079 | 8.65E-06 | 0.0416 |
| 93 | cg10939667 | *ESR1* | 6 | -0.0037 | -0.0116 | 8.73E-06 | 0.0416 |
| 94 | cg20003138 | *UTP20* | 12 | 0.0042 | 0.0101 | 8.75E-06 | 0.0416 |
| 95 | cg05745100 | *BRSK2* | 11 | -0.0191 | -0.0053 | 8.81E-06 | 0.0416 |
| 96 | cg04657588 | *GPRC5C* | 17 | -0.0002 | 0.0062 | 9.01E-06 | 0.0421 |
| 97 | cg02787917 |  | 2 | 0.0063 | 0.0090 | 9.40E-06 | 0.0435 |
| 98 | cg15644742 | *PEX10* | 1 | 0.0001 | 0.0014 | 9.58E-06 | 0.0439 |
| 99 | cg25331919 | *LIPG* | 18 | -0.0092 | -0.0158 | 9.82E-06 | 0.0442 |
| 100 | cg14329833 | *TMEM140* | 7 | -0.0176 | -0.0206 | 9.84E-06 | 0.0442 |
| 101 | cg22748740 | *ZNF311* | 6 | -0.0003 | 0.0039 | 9.92E-06 | 0.0442 |
| 102 | cg18238366 | *SCAND1* | 20 | -0.0003 | 0.0009 | 1.02E-05 | 0.0450 |
| 103 | cg18505401 | *TMEM196* | 7 | -0.0003 | 0.0050 | 1.05E-05 | 0.0460 |
| 104 | cg17388712 |  | 7 | -0.0092 | -0.0137 | 1.11E-05 | 0.0480 |
