# Supplementary material for: Prenatal cannabis exposure is associated with alterations in offspring DNA methylation at genes involved in neurodevelopment, across the life course
Source: Mol Psychiatry. 2024 Sep 14;30(4):1418–29. doi: 10.1038/s41380-024-02752-w (PMC11919715; doi:10.1038/s41380-024-02752-w)
Supplement: Supplementary file 3 — Supplementary Table 2 [file 41380_2024_2752_MOESM3_ESM.docx]

**Supplementary Table 2**

All significantly differentially methylated CpG sites in response to PCE at 7 y (ALSPAC)

| **Rank** | **IlmnID** | **Gene Name** | **CHR** | **Beta difference** | **logFC** | **P.Value** | **adj.P.Val** |
| --- | --- | --- | --- | --- | --- | --- | --- |
| 1 | cg10170214 | *LZTS2* | 10 | -0.0009 | -0.0092 | 9.12E-10 | 0.0004 |
| 2 | cg25208479 |  | 15 | 0.0017 | 0.0115 | 1.09E-07 | 0.0254 |
| 3 | cg16028064 | *BPTF* | 17 | -0.0002 | 0.0020 | 2.39E-07 | 0.0254 |
| 4 | cg02850468 | *NPSR1* | 7 | -0.0403 | -0.0222 | 2.53E-07 | 0.0254 |
| 5 | cg23992470 | *GAK* | 4 | -0.0033 | -0.0104 | 5.15E-07 | 0.0354 |
| 6 | cg10872815 | *PDE7B* | 6 | -0.0069 | -0.0109 | 5.39E-07 | 0.0354 |
| 7 | cg00731404 | *MTIF3* | 13 | 0.0000 | 0.0032 | 6.65E-07 | 0.0354 |
| 8 | cg11681126 | *ZNF32* | 10 | 0.0006 | 0.0114 | 7.27E-07 | 0.0354 |
| 9 | cg23938542 | *CRIP2* | 14 | 0.0007 | 0.0035 | 7.58E-07 | 0.0354 |
| 10 | cg00646883 | *COX18* | 4 | 0.0019 | 0.0111 | 8.82E-07 | 0.0354 |
| 11 | cg17721710 | *SLC30A10* | 1 | 0.0003 | 0.0024 | 9.32E-07 | 0.0354 |
| 12 | cg14344315 |  | 15 | 0.0034 | 0.0027 | 1.08E-06 | 0.0354 |
| 13 | cg23762037 | *TUBGCP6* | 22 | 0.0001 | 0.0016 | 1.18E-06 | 0.0354 |
| 14 | cg19906737 | *C8orf41* | 8 | -0.0001 | 0.0048 | 1.37E-06 | 0.0354 |
| 15 | cg13770088 | *PRRG4* | 11 | -0.0001 | 0.0021 | 1.53E-06 | 0.0354 |
| 16 | cg18654873 | *MAP3K7* | 6 | 0.0002 | 0.0038 | 1.53E-06 | 0.0354 |
| 17 | cg18262051 | *MEPCE* | 7 | 0.0046 | 0.0041 | 1.65E-06 | 0.0354 |
| 18 | cg04010471 |  | 13 | 0.0001 | 0.0020 | 1.65E-06 | 0.0354 |
| 19 | cg20514239 | *RBP2* | 3 | -0.0174 | -0.0245 | 1.67E-06 | 0.0354 |
| 20 | cg18112005 | *C14orf80* | 14 | 0.0003 | 0.0043 | 1.68E-06 | 0.0354 |
| 21 | cg23690444 |  | 14 | -0.0005 | 0.0049 | 1.70E-06 | 0.0354 |
| 22 | cg10101749 | *MFSD2A* | 1 | -0.0003 | 0.0101 | 1.80E-06 | 0.0354 |
| 23 | cg21241291 | *C14orf80* | 14 | 0.0003 | 0.0045 | 1.84E-06 | 0.0354 |
| 24 | cg09155701 | *ARL10* | 5 | -0.0002 | 0.0016 | 1.95E-06 | 0.0354 |
| 25 | cg24635468 | *NT5E* | 6 | 0.0000 | 0.0035 | 2.07E-06 | 0.0362 |
| 26 | cg09613161 | *COBL* | 7 | -0.0005 | 0.0035 | 2.42E-06 | 0.0394 |
| 27 | cg06577045 | *POPDC3* | 6 | -0.0002 | 0.0018 | 2.60E-06 | 0.0398 |
| 28 | cg10825876 | *PLA2R1* | 2 | 0.0003 | 0.0017 | 2.61E-06 | 0.0398 |
| 29 | cg18576588 | *CRIP2* | 14 | 0.0002 | 0.0040 | 2.89E-06 | 0.0427 |
| 30 | cg19302295 | *PRKACA* | 19 | 0.0000 | 0.0019 | 3.53E-06 | 0.0457 |
| 31 | cg25933015 | *BBS9* | 7 | 0.0017 | -0.0082 | 3.58E-06 | 0.0457 |
| 32 | cg11302943 | *CRTAC1* | 10 | -0.0005 | 0.0017 | 3.65E-06 | 0.0457 |
| 33 | cg19141861 | *COQ5* | 12 | -0.0001 | 0.0031 | 3.88E-06 | 0.0457 |
| 34 | cg13280063 |  | 6 | -0.0213 | -0.0150 | 3.94E-06 | 0.0457 |
| 35 | cg18679920 | *C14orf80* | 14 | 0.0000 | 0.0041 | 3.96E-06 | 0.0457 |
| 36 | cg00944001 | *VAC14* | 16 | -0.0037 | -0.0142 | 3.97E-06 | 0.0457 |
