# Supplementary material for: Prenatal cannabis exposure is associated with alterations in offspring DNA methylation at genes involved in neurodevelopment, across the life course
Source: Mol Psychiatry. 2024 Sep 14;30(4):1418–29. doi: 10.1038/s41380-024-02752-w (PMC11919715; doi:10.1038/s41380-024-02752-w)
Supplement: Supplementary file 4 — Supplementary Table 3 [file 41380_2024_2752_MOESM4_ESM.docx]

**Supplementary Table 3**

All significantly differentially methylated CpG sites in response to PCE at 15-17 y (ALSPAC)

|  |  |  |  |  |  |  |  |
| --- | --- | --- | --- | --- | --- | --- | --- |
| **Rank** | **IlmnID** | **Gene Name** | **CHR** | **Beta difference** | **logFC** | **P.Value** | **adj.P.Val** |
| 1 | cg13799287 | *WAC* | 10 | 0.0001 | 0.0063 | 2.20E-10 | 5.16E-05 |
| 2 | cg20249919 | *PCSK6* | 15 | 0.0024 | 0.0089 | 2.84E-10 | 5.16E-05 |
| 3 | cg14316565 | *CAT* | 11 | 0.0023 | 0.0077 | 3.28E-10 | 5.16E-05 |
| 4 | cg14703784 | *RASA3* | 13 | 0.0002 | -0.0101 | 6.50E-10 | 7.68E-05 |
| 5 | cg05165940 |  | 2 | 0.0049 | 0.0220 | 8.29E-10 | 7.83E-05 |
| 6 | cg01450600 | *FAM160B1* | 10 | -0.0069 | -0.0183 | 5.36E-09 | 0.0004 |
| 7 | cg13692446 |  | 13 | 0.0009 | 0.0070 | 2.16E-08 | 0.0013 |
| 8 | cg21108767 | *PILRB* | 7 | -0.0010 | 0.0092 | 3.32E-08 | 0.0017 |
| 9 | cg10888941 |  | 2 | 0.0048 | 0.0068 | 3.99E-08 | 0.0019 |
| 10 | cg14283922 | *ACBD3* | 1 | 0.0017 | 0.0088 | 5.44E-08 | 0.0022 |
| 11 | cg15994321 | *ATF6* | 1 | 0.0002 | 0.0033 | 5.73E-08 | 0.0022 |
| 12 | cg03886242 | *NFE2L3* | 7 | 0.0005 | 0.0017 | 5.96E-08 | 0.0022 |
| 13 | cg16528511 | *RIPPLY2* | 6 | 0.0032 | 0.0048 | 7.49E-08 | 0.0025 |
| 14 | cg25559490 | *WRNIP1* | 6 | 0.0004 | 0.0066 | 9.74E-08 | 0.0029 |
| 15 | cg10416994 | *FBXO21* | 12 | -0.0012 | 0.0056 | 1.04E-07 | 0.0029 |
| 16 | cg11443159 | *IRGQ* | 19 | 0.0061 | 0.0068 | 1.09E-07 | 0.0029 |
| 17 | cg16174609 | *R3HDML* | 20 | 0.0005 | -0.0145 | 1.10E-07 | 0.0029 |
| 18 | cg23494140 | *EDNRB* | 13 | 0.0021 | 0.0068 | 1.27E-07 | 0.0032 |
| 19 | cg24635468 | *NT5E* | 6 | -0.0003 | 0.0020 | 1.36E-07 | 0.0032 |
| 20 | cg07479092 | *PLK2* | 5 | 0.0037 | 0.0078 | 1.46E-07 | 0.0033 |
| 21 | cg07173670 | *SPG7* | 16 | 0.0000 | 0.0046 | 1.52E-07 | 0.0033 |
| 22 | cg00475161 | *KDELR1* | 19 | 0.0008 | 0.0070 | 2.01E-07 | 0.0041 |
| 23 | cg22456785 |  | 19 | 0.0028 | 0.0051 | 2.31E-07 | 0.0045 |
| 24 | cg10598433 | *EBF3* | 10 | -0.0059 | -0.0139 | 2.47E-07 | 0.0045 |
| 25 | cg26296762 |  | 1 | 0.0037 | 0.0074 | 2.54E-07 | 0.0045 |
| 26 | cg18041814 | *CPNE5* | 6 | -0.0039 | -0.0180 | 3.17E-07 | 0.0045 |
| 27 | cg06699063 | *C17orf70* | 17 | 0.0001 | 0.0078 | 3.65E-07 | 0.0045 |
| 28 | cg27505538 | *STARD5* | 15 | 0.0013 | 0.0048 | 4.31E-07 | 0.0045 |
| 29 | cg02104993 | *TOM1L1* | 17 | 0.0001 | 0.0070 | 4.32E-07 | 0.0045 |
| 30 | cg07336097 | *NFKBIL2* | 8 | 0.0000 | 0.0053 | 4.52E-07 | 0.0045 |
| 31 | cg07132086 | *PLEKHA2* | 8 | -0.0016 | -0.0106 | 4.62E-07 | 0.0045 |
| 32 | cg15364276 | *MON2* | 12 | 0.0011 | 0.0033 | 4.63E-07 | 0.0045 |
| 33 | cg16583330 | *OSCP1* | 1 | 0.0039 | 0.0122 | 4.82E-07 | 0.0045 |
| 34 | cg23156916 |  | 8 | -0.0004 | 0.0070 | 5.04E-07 | 0.0045 |
| 35 | cg25880288 | *MIB2* | 1 | 0.0006 | 0.0116 | 5.29E-07 | 0.0045 |
| 36 | cg20239160 | *JUP* | 17 | -0.0035 | -0.0145 | 5.45E-07 | 0.0045 |
| 37 | cg09068665 | *CACNA1C* | 12 | 0.0001 | 0.0070 | 5.51E-07 | 0.0045 |
| 38 | cg20488617 | *SNAI1* | 20 | 0.0003 | 0.0066 | 5.56E-07 | 0.0045 |
| 39 | cg24869475 | *LOC401052* | 3 | 0.0002 | 0.0072 | 5.65E-07 | 0.0045 |
| 40 | cg00924357 | *MKLN1* | 7 | 0.0001 | 0.0061 | 5.68E-07 | 0.0045 |
| 41 | cg18259915 | *LSM14B* | 20 | 0.0013 | 0.0076 | 5.76E-07 | 0.0045 |
| 42 | cg12214665 | *TNFAIP3* | 6 | 0.0001 | 0.0075 | 5.82E-07 | 0.0045 |
| 43 | cg17982643 | *C11orf71* | 11 | 0.0002 | 0.0059 | 5.92E-07 | 0.0045 |
| 44 | cg04736260 | *SELV* | 19 | 0.0007 | 0.0070 | 6.04E-07 | 0.0045 |
| 45 | cg25057861 | *LRRC20* | 10 | 0.0002 | 0.0058 | 6.14E-07 | 0.0045 |
| 46 | cg22719138 | *PDCD2L* | 19 | 0.0000 | 0.0064 | 6.27E-07 | 0.0045 |
| 47 | cg03603808 | *CCNE1* | 19 | 0.0004 | 0.0059 | 6.75E-07 | 0.0045 |
| 48 | cg25007572 | *TMEM223* | 11 | -0.0001 | 0.0071 | 6.76E-07 | 0.0045 |
| 49 | cg15966876 |  | 18 | -0.0003 | 0.0068 | 6.84E-07 | 0.0045 |
| 50 | cg03520976 | *RBM20* | 10 | 0.0002 | 0.0058 | 6.87E-07 | 0.0045 |
| 51 | cg10396357 | *TFAP4* | 16 | 0.0002 | 0.0045 | 6.91E-07 | 0.0045 |
| 52 | cg22746256 | *EGR2* | 10 | 0.0019 | 0.0077 | 6.98E-07 | 0.0045 |
| 53 | cg03156032 | *TAOK1* | 17 | 0.0003 | 0.0078 | 7.11E-07 | 0.0045 |
| 54 | cg04313601 | *SBF2* | 11 | 0.0000 | 0.0054 | 7.41E-07 | 0.0045 |
| 55 | cg25385412 | *ELOVL1* | 1 | 0.0004 | 0.0057 | 7.42E-07 | 0.0045 |
| 56 | cg14677317 | *ARHGEF18* | 19 | -0.0001 | 0.0066 | 7.45E-07 | 0.0045 |
| 57 | cg18318639 | *MOCS2* | 5 | 0.0001 | 0.0058 | 7.69E-07 | 0.0045 |
| 58 | cg17476405 | *MICA* | 6 | 0.0009 | 0.0015 | 7.88E-07 | 0.0045 |
| 59 | cg09395318 | *GLB1L3* | 11 | 0.0000 | 0.0069 | 7.96E-07 | 0.0045 |
| 60 | cg01697541 | *TMEM97* | 17 | 0.0003 | 0.0060 | 8.01E-07 | 0.0045 |
| 61 | cg12810313 | *MSRA* | 8 | 0.0005 | 0.0076 | 8.26E-07 | 0.0045 |
| 62 | cg14725215 | *PELP1* | 17 | 0.0019 | 0.0062 | 8.31E-07 | 0.0045 |
| 63 | cg10418716 | *ANKRD13A* | 12 | 0.0003 | 0.0065 | 8.37E-07 | 0.0045 |
| 64 | cg16857858 | *HOXA10* | 7 | 0.0001 | 0.0060 | 8.38E-07 | 0.0045 |
| 65 | cg19208700 | *RNFT2* | 12 | 0.0005 | 0.0061 | 8.46E-07 | 0.0045 |
| 66 | cg26787998 | *TAPBP* | 6 | 0.0013 | 0.0062 | 8.47E-07 | 0.0045 |
| 67 | cg00390769 | *MTERF* | 7 | -0.0001 | 0.0068 | 8.55E-07 | 0.0045 |
| 68 | cg27299538 | *PTEN* | 10 | 0.0004 | 0.0059 | 8.57E-07 | 0.0045 |
| 69 | cg24976104 | *PRR5* | 22 | 0.0005 | 0.0062 | 8.63E-07 | 0.0045 |
| 70 | cg15705054 | *PBXIP1* | 1 | 0.0011 | 0.0064 | 8.65E-07 | 0.0045 |
| 71 | cg22157823 | *SCAP* | 3 | 0.0002 | 0.0058 | 8.76E-07 | 0.0045 |
| 72 | cg00809888 | *NAAA* | 4 | 0.0005 | 0.0069 | 8.77E-07 | 0.0045 |
| 73 | cg15996043 | *SMOC1* | 14 | 0.0005 | 0.0028 | 8.83E-07 | 0.0045 |
| 74 | cg16832267 | *KCNS3* | 2 | -0.0021 | 0.0135 | 8.83E-07 | 0.0045 |
| 75 | cg26150754 |  | 1 | -0.0004 | 0.0057 | 8.92E-07 | 0.0045 |
| 76 | cg10310846 | *WAC* | 10 | 0.0007 | 0.0059 | 9.34E-07 | 0.0045 |
| 77 | cg24166112 | *TRIM47* | 17 | 0.0005 | 0.0056 | 9.45E-07 | 0.0045 |
| 78 | ch.9.2215767R | *FREQ* | 9 | -0.0010 | 0.0057 | 9.48E-07 | 0.0045 |
| 79 | cg26147372 | *QRICH1* | 3 | 0.0002 | 0.0058 | 9.64E-07 | 0.0045 |
| 80 | cg10106804 | *GJA4* | 1 | 0.0003 | 0.0082 | 9.84E-07 | 0.0045 |
| 81 | cg15807973 | *WAPAL* | 10 | 0.0004 | 0.0068 | 9.96E-07 | 0.0045 |
| 82 | cg15143799 | *ZNF248* | 10 | 0.0009 | 0.0066 | 1.02E-06 | 0.0045 |
| 83 | cg23092250 | *ASPHD2* | 22 | 0.0000 | 0.0059 | 1.02E-06 | 0.0045 |
| 84 | cg17334018 | *UNC5C* | 4 | -0.0005 | 0.0099 | 1.02E-06 | 0.0045 |
| 85 | cg22113930 | *ATP10A* | 15 | 0.0005 | 0.0064 | 1.02E-06 | 0.0045 |
| 86 | cg22886405 | *GTF2IRD2B* | 7 | 0.0002 | 0.0056 | 1.03E-06 | 0.0045 |
| 87 | cg15831613 | *ATF3* | 1 | 0.0002 | 0.0071 | 1.03E-06 | 0.0045 |
| 88 | cg01850934 | *VTI1B* | 14 | -0.0004 | 0.0060 | 1.05E-06 | 0.0045 |
| 89 | cg14397459 | *USP15* | 12 | 0.0001 | 0.0066 | 1.05E-06 | 0.0045 |
| 90 | cg06751243 |  | 20 | 0.0077 | 0.0164 | 1.07E-06 | 0.0045 |
| 91 | cg05469916 | *NCAPG2* | 7 | -0.0005 | 0.0090 | 1.07E-06 | 0.0045 |
| 92 | cg00424800 | *GPR160* | 3 | -0.0023 | 0.0110 | 1.09E-06 | 0.0045 |
| 93 | cg26388450 | *PMP22* | 17 | 0.0002 | 0.0066 | 1.12E-06 | 0.0045 |
| 94 | cg15042811 | *FREM3* | 4 | 0.0023 | 0.0022 | 1.12E-06 | 0.0045 |
| 95 | cg10397063 | *BCLAF1* | 6 | -0.0001 | 0.0062 | 1.13E-06 | 0.0045 |
| 96 | cg04793821 | *KDM3A* | 2 | 0.0001 | 0.0058 | 1.16E-06 | 0.0045 |
| 97 | cg15754548 | *SLC25A35* | 17 | -0.0001 | 0.0019 | 1.18E-06 | 0.0045 |
| 98 | cg23069877 |  | 2 | -0.0289 | -0.0171 | 1.18E-06 | 0.0045 |
| 99 | cg18927255 | *SMEK1* | 14 | 0.0002 | 0.0061 | 1.19E-06 | 0.0045 |
| 100 | cg11356705 | *NUP54* | 4 | 0.0004 | 0.0062 | 1.19E-06 | 0.0045 |
| 101 | cg07595113 | *MYBL1* | 8 | 0.0002 | 0.0065 | 1.20E-06 | 0.0045 |
| 102 | cg10472244 | *SELK* | 3 | 0.0002 | 0.0067 | 1.21E-06 | 0.0045 |
| 103 | cg18115660 | *RGS19* | 20 | 0.0004 | 0.0065 | 1.21E-06 | 0.0045 |
| 104 | cg07924819 | *ATF6* | 1 | 0.0002 | 0.0038 | 1.21E-06 | 0.0045 |
| 105 | cg05427356 | *RPL8* | 8 | 0.0003 | 0.0060 | 1.23E-06 | 0.0045 |
| 106 | cg26538529 | *EPHA4* | 2 | 0.0002 | 0.0055 | 1.23E-06 | 0.0045 |
| 107 | cg09388080 | *TBC1D16* | 17 | 0.0009 | -0.0082 | 1.23E-06 | 0.0045 |
| 108 | cg09719331 | *ZSWIM3* | 20 | 0.0012 | 0.0099 | 1.23E-06 | 0.0045 |
| 109 | cg24102761 | *CASP7* | 10 | 0.0013 | 0.0060 | 1.24E-06 | 0.0045 |
| 110 | cg16681130 | *PCCA* | 13 | -0.0002 | 0.0060 | 1.25E-06 | 0.0045 |
| 111 | cg07401045 | *LOC93622* | 4 | 0.0017 | 0.0069 | 1.26E-06 | 0.0045 |
| 112 | cg06087029 | *ATRIP* | 3 | 0.0008 | 0.0064 | 1.26E-06 | 0.0045 |
| 113 | cg24592962 | *AS3MT* | 10 | 0.0036 | 0.0071 | 1.28E-06 | 0.0045 |
| 114 | cg12317151 | *LPIN2* | 18 | 0.0001 | 0.0062 | 1.30E-06 | 0.0045 |
| 115 | cg07038191 | *KIF22* | 16 | -0.0002 | 0.0057 | 1.30E-06 | 0.0045 |
| 116 | cg26566224 | *MSRA* | 8 | -0.0030 | -0.0117 | 1.31E-06 | 0.0045 |
| 117 | cg00076538 | *EGFLAM* | 5 | 0.0008 | 0.0072 | 1.31E-06 | 0.0045 |
| 118 | cg02189786 | *THAP11* | 16 | 0.0001 | 0.0097 | 1.31E-06 | 0.0045 |
| 119 | cg11612592 | *CDC27* | 17 | 0.0005 | 0.0060 | 1.33E-06 | 0.0045 |
| 120 | cg15034345 | *SOX11* | 2 | 0.0000 | 0.0066 | 1.33E-06 | 0.0045 |
| 121 | cg02738049 | *ZSCAN29* | 15 | 0.0001 | 0.0065 | 1.33E-06 | 0.0045 |
| 122 | cg26677084 | *ST6GALNAC2* | 17 | 0.0000 | 0.0060 | 1.34E-06 | 0.0045 |
| 123 | cg27305704 |  | 1 | -0.0008 | 0.0059 | 1.34E-06 | 0.0045 |
| 124 | cg03740159 | *SRPK2* | 7 | -0.0002 | 0.0061 | 1.35E-06 | 0.0045 |
| 125 | cg25678491 | *TMTC4* | 13 | -0.0004 | 0.0074 | 1.35E-06 | 0.0045 |
| 126 | cg03578822 | *WDR1* | 4 | 0.0001 | 0.0056 | 1.36E-06 | 0.0045 |
| 127 | cg01758856 | *TBCD* | 17 | -0.0103 | -0.0123 | 1.39E-06 | 0.0045 |
| 128 | cg26464887 | *LOC100271722* | 22 | 0.0007 | 0.0063 | 1.42E-06 | 0.0045 |
| 129 | cg00852041 | *ZFYVE28* | 4 | 0.0000 | 0.0056 | 1.44E-06 | 0.0045 |
| 130 | cg25654957 | *BLCAP* | 20 | 0.0003 | 0.0073 | 1.47E-06 | 0.0045 |
| 131 | cg02226779 | *EWSR1* | 22 | 0.0002 | 0.0062 | 1.49E-06 | 0.0045 |
| 132 | cg19758151 |  | 17 | 0.0010 | 0.0087 | 1.50E-06 | 0.0045 |
| 133 | cg20718724 | *P2RY2* | 11 | 0.0000 | 0.0069 | 1.50E-06 | 0.0045 |
| 134 | cg26319343 | *SAFB2* | 19 | -0.0001 | 0.0067 | 1.50E-06 | 0.0045 |
| 135 | cg24217936 | *CPEB2* | 4 | 0.0001 | 0.0060 | 1.52E-06 | 0.0045 |
| 136 | cg18513123 | *XRRA1* | 11 | 0.0000 | 0.0099 | 1.53E-06 | 0.0045 |
| 137 | cg07752304 | *ARID3A* | 19 | -0.0001 | 0.0062 | 1.54E-06 | 0.0045 |
| 138 | cg08457790 |  | 6 | 0.0001 | 0.0110 | 1.55E-06 | 0.0045 |
| 139 | cg06321345 | *FAM5B* | 1 | -0.0004 | 0.0061 | 1.56E-06 | 0.0045 |
| 140 | cg12361311 | *TRPC4AP* | 20 | 0.0002 | 0.0058 | 1.56E-06 | 0.0045 |
| 141 | cg22508203 |  | 13 | -0.0004 | 0.0062 | 1.59E-06 | 0.0045 |
| 142 | cg22475904 | *PCGF1* | 2 | 0.0000 | 0.0095 | 1.59E-06 | 0.0045 |
| 143 | cg00161970 |  | 1 | 0.0026 | 0.0081 | 1.60E-06 | 0.0045 |
| 144 | cg19895882 | *KIAA0564* | 13 | 0.0000 | 0.0059 | 1.61E-06 | 0.0045 |
| 145 | cg02974499 | *FAU* | 11 | 0.0002 | 0.0066 | 1.61E-06 | 0.0045 |
| 146 | cg10506549 | *TRIM39* | 6 | 0.0000 | 0.0068 | 1.61E-06 | 0.0045 |
| 147 | cg24428325 |  | 6 | 0.0002 | 0.0052 | 1.62E-06 | 0.0045 |
| 148 | cg23893802 | *ZNF425* | 7 | -0.0001 | 0.0060 | 1.64E-06 | 0.0045 |
| 149 | cg26037945 | *KCNIP2* | 10 | 0.0001 | 0.0061 | 1.65E-06 | 0.0045 |
| 150 | cg18000924 | *NOP58* | 2 | -0.0003 | 0.0077 | 1.65E-06 | 0.0045 |
| 151 | cg27075171 | *GLO1* | 6 | -0.0001 | 0.0060 | 1.66E-06 | 0.0045 |
| 152 | cg06978273 | *TACR2* | 10 | 0.0005 | 0.0059 | 1.66E-06 | 0.0045 |
| 153 | cg11063167 | *TXN2* | 22 | 0.0011 | 0.0059 | 1.66E-06 | 0.0045 |
| 154 | cg21971035 | *HCCA2* | 11 | 0.0001 | 0.0044 | 1.67E-06 | 0.0045 |
| 155 | cg27009828 | *EEF2* | 19 | 0.0007 | 0.0066 | 1.68E-06 | 0.0045 |
| 156 | cg24606935 | *HCK* | 20 | -0.0002 | 0.0058 | 1.70E-06 | 0.0045 |
| 157 | cg19168326 | *C21orf45* | 21 | -0.0001 | 0.0059 | 1.71E-06 | 0.0045 |
| 158 | cg17707966 | *ZNF623* | 8 | 0.0002 | 0.0059 | 1.71E-06 | 0.0045 |
| 159 | cg11155915 | *SKI* | 1 | -0.0004 | 0.0059 | 1.71E-06 | 0.0045 |
| 160 | cg20139898 | *PDIK1L* | 1 | 0.0000 | 0.0054 | 1.71E-06 | 0.0045 |
| 161 | cg22836535 | *C14orf80* | 14 | 0.0002 | 0.0055 | 1.71E-06 | 0.0045 |
| 162 | cg07796735 | *KCNH3* | 12 | 0.0001 | 0.0075 | 1.72E-06 | 0.0045 |
| 163 | cg24532898 | *TLK1* | 2 | -0.0001 | 0.0061 | 1.74E-06 | 0.0045 |
| 164 | cg01841828 | *CHTF8* | 16 | 0.0011 | 0.0028 | 1.74E-06 | 0.0045 |
| 165 | cg01716380 | *NAA16* | 13 | -0.0007 | 0.0060 | 1.76E-06 | 0.0045 |
| 166 | cg08331842 | *HEXA* | 15 | 0.0003 | 0.0100 | 1.77E-06 | 0.0045 |
| 167 | cg01368872 | *RCCD1* | 15 | 0.0001 | 0.0060 | 1.78E-06 | 0.0045 |
| 168 | cg04995722 | *NFE2L3* | 7 | 0.0005 | 0.0019 | 1.79E-06 | 0.0045 |
| 169 | cg00470794 | *CHFR* | 12 | 0.0008 | 0.0061 | 1.83E-06 | 0.0045 |
| 170 | cg14439353 | *EEF1E1* | 6 | 0.0014 | 0.0044 | 1.84E-06 | 0.0045 |
| 171 | cg09107957 | *C1orf131* | 1 | -0.0009 | 0.0057 | 1.84E-06 | 0.0045 |
| 172 | cg16587158 | *EIF2B2* | 14 | 0.0002 | 0.0053 | 1.84E-06 | 0.0045 |
| 173 | cg23741639 |  | 11 | -0.0004 | -0.0111 | 1.84E-06 | 0.0045 |
| 174 | cg22710840 | *FAM72D* | 1 | 0.0002 | 0.0051 | 1.87E-06 | 0.0045 |
| 175 | cg24073340 | *RIC8A* | 11 | -0.0001 | 0.0067 | 1.88E-06 | 0.0045 |
| 176 | cg05304177 | *AMOTL2* | 3 | -0.0009 | 0.0067 | 1.88E-06 | 0.0045 |
| 177 | cg00950958 | *TOX2* | 20 | -0.0001 | 0.0060 | 1.90E-06 | 0.0045 |
| 178 | cg17303459 | *NDUFA3* | 19 | -0.0001 | 0.0061 | 1.91E-06 | 0.0045 |
| 179 | cg08204131 | *SPTBN4* | 19 | 0.0001 | 0.0055 | 1.92E-06 | 0.0045 |
| 180 | cg25722198 | *PARP1* | 1 | -0.0002 | 0.0058 | 1.93E-06 | 0.0045 |
| 181 | cg14219291 | *FAM175B* | 10 | -0.0008 | 0.0075 | 1.94E-06 | 0.0045 |
| 182 | cg18576588 | *CRIP2* | 14 | -0.0001 | 0.0026 | 1.94E-06 | 0.0045 |
| 183 | cg10293093 |  | 6 | 0.0003 | 0.0062 | 1.95E-06 | 0.0045 |
| 184 | cg05357320 | *ZNF317* | 19 | -0.0003 | 0.0068 | 1.95E-06 | 0.0045 |
| 185 | cg10178261 | *IMP3* | 15 | 0.0001 | 0.0077 | 1.96E-06 | 0.0045 |
| 186 | cg09096528 | *CDK4* | 12 | -0.0002 | 0.0057 | 1.97E-06 | 0.0045 |
| 187 | cg00584416 | *RSBN1* | 1 | 0.0000 | 0.0056 | 1.98E-06 | 0.0045 |
| 188 | cg15465548 | *KIF23* | 15 | -0.0001 | 0.0081 | 1.99E-06 | 0.0045 |
| 189 | cg19786784 | *ACACA* | 17 | -0.0002 | 0.0064 | 2.01E-06 | 0.0045 |
| 190 | cg07251603 | *FLNB* | 3 | 0.0005 | 0.0071 | 2.04E-06 | 0.0046 |
| 191 | cg17189748 | *C3orf10* | 3 | 0.0003 | 0.0059 | 2.05E-06 | 0.0046 |
| 192 | cg06851900 | *ATP1A1* | 1 | -0.0002 | 0.0053 | 2.06E-06 | 0.0046 |
| 193 | cg21820889 | *KPNA2* | 17 | 0.0008 | 0.0037 | 2.06E-06 | 0.0046 |
| 194 | cg15906151 | *SETDB2* | 13 | -0.0001 | 0.0062 | 2.06E-06 | 0.0046 |
| 195 | cg16452756 | *C5orf41* | 5 | 0.0000 | 0.0053 | 2.07E-06 | 0.0046 |
| 196 | cg17757602 |  | 5 | 0.0002 | 0.0067 | 2.11E-06 | 0.0046 |
| 197 | cg16841147 | *GSS* | 20 | 0.0000 | 0.0106 | 2.12E-06 | 0.0046 |
| 198 | cg21799227 | *COG1* | 17 | 0.0004 | 0.0060 | 2.12E-06 | 0.0046 |
| 199 | cg06401851 | *FUS* | 16 | -0.0002 | 0.0062 | 2.13E-06 | 0.0046 |
| 200 | cg26692046 | *TBC1D20* | 20 | 0.0002 | 0.0075 | 2.13E-06 | 0.0046 |
| 201 | cg14538374 | *ALDH18A1* | 10 | -0.0001 | 0.0055 | 2.14E-06 | 0.0046 |
| 202 | cg22797900 | *SCRT2* | 20 | 0.0024 | 0.0073 | 2.16E-06 | 0.0046 |
| 203 | cg11125146 | *CITED2* | 6 | 0.0002 | 0.0040 | 2.17E-06 | 0.0046 |
| 204 | cg23868899 | *FBXO32* | 8 | -0.0002 | 0.0065 | 2.19E-06 | 0.0046 |
| 205 | cg27425452 | *PARK2* | 6 | 0.0001 | 0.0057 | 2.19E-06 | 0.0046 |
| 206 | cg06602498 | *RABGEF1* | 7 | 0.0003 | 0.0066 | 2.21E-06 | 0.0046 |
| 207 | cg10330832 | *CNST* | 1 | -0.0002 | 0.0059 | 2.23E-06 | 0.0046 |
| 208 | cg22562194 | *TTYH2* | 17 | 0.0012 | 0.0062 | 2.23E-06 | 0.0046 |
| 209 | cg26102512 | *WDR81* | 17 | 0.0024 | 0.0085 | 2.23E-06 | 0.0046 |
| 210 | cg17307196 | *STAM* | 10 | 0.0015 | 0.0042 | 2.24E-06 | 0.0046 |
| 211 | cg19233769 | *ARF1* | 1 | 0.0005 | 0.0055 | 2.26E-06 | 0.0046 |
| 212 | cg00680003 | *GNL1* | 6 | 0.0045 | 0.0117 | 2.31E-06 | 0.0046 |
| 213 | cg19334895 | *UPF1* | 19 | -0.0002 | 0.0078 | 2.31E-06 | 0.0046 |
| 214 | cg03433712 | *VPS13C* | 15 | -0.0005 | 0.0114 | 2.31E-06 | 0.0046 |
| 215 | cg01533539 | *SPATA2L* | 16 | 0.0004 | 0.0061 | 2.32E-06 | 0.0046 |
| 216 | cg00773370 | *SCNN1A* | 12 | -0.0007 | -0.0082 | 2.33E-06 | 0.0046 |
| 217 | cg14375282 |  | 17 | -0.0011 | 0.0075 | 2.34E-06 | 0.0046 |
| 218 | cg20655648 | *IMP3* | 15 | 0.0000 | 0.0059 | 2.36E-06 | 0.0046 |
| 219 | cg07645718 | *TCFL5* | 20 | 0.0004 | 0.0018 | 2.37E-06 | 0.0046 |
| 220 | cg27067269 | *ZNF488* | 10 | -0.0001 | 0.0062 | 2.37E-06 | 0.0046 |
| 221 | cg18881377 | *C12orf62* | 12 | 0.0002 | 0.0061 | 2.39E-06 | 0.0046 |
| 222 | cg21764190 | *PAX6* | 11 | 0.0000 | 0.0061 | 2.39E-06 | 0.0046 |
| 223 | cg05252161 | *C14orf80* | 14 | 0.0001 | 0.0023 | 2.42E-06 | 0.0047 |
| 224 | cg23455341 | *TRIM25* | 17 | -0.0001 | 0.0062 | 2.46E-06 | 0.0047 |
| 225 | cg13973620 | *RGL2* | 6 | 0.0017 | 0.0134 | 2.48E-06 | 0.0047 |
| 226 | cg27544191 | *SBF1* | 22 | -0.0003 | 0.0061 | 2.52E-06 | 0.0048 |
| 227 | cg00537387 | *DOM3Z* | 6 | 0.0000 | 0.0058 | 2.53E-06 | 0.0048 |
| 228 | cg16561266 | *LOC146880* | 17 | -0.0008 | 0.0066 | 2.54E-06 | 0.0048 |
| 229 | cg22506605 | *SNX21* | 20 | 0.0052 | 0.0063 | 2.54E-06 | 0.0048 |
| 230 | cg01993865 | *DSTN* | 20 | 0.0002 | 0.0070 | 2.55E-06 | 0.0048 |
| 231 | cg06128055 | *HECTD2* | 10 | -0.0001 | 0.0067 | 2.56E-06 | 0.0048 |
| 232 | cg16863990 | *THSD1* | 13 | 0.0008 | 0.0019 | 2.58E-06 | 0.0048 |
| 233 | cg03993376 | *LOC100268168* | 5 | -0.0013 | 0.0055 | 2.60E-06 | 0.0048 |
| 234 | cg12878555 | *MLF2* | 12 | 0.0008 | 0.0057 | 2.60E-06 | 0.0048 |
| 235 | cg13017478 | *IL17RD* | 3 | -0.0007 | 0.0056 | 2.63E-06 | 0.0048 |
| 236 | cg05250874 | *MLLT6* | 17 | -0.0004 | 0.0079 | 2.63E-06 | 0.0048 |
| 237 | cg22788080 | *ERCC6* | 10 | -0.0001 | 0.0063 | 2.64E-06 | 0.0048 |
| 238 | cg06140764 | *ABCF1* | 6 | -0.0002 | 0.0067 | 2.64E-06 | 0.0048 |
| 239 | cg17171801 | *PMP22* | 17 | 0.0000 | 0.0059 | 2.68E-06 | 0.0048 |
| 240 | cg18430156 | *FAM171B* | 2 | 0.0010 | 0.0067 | 2.70E-06 | 0.0049 |
| 241 | cg24171907 | *CNRIP1* | 2 | -0.0003 | 0.0038 | 2.70E-06 | 0.0049 |
| 242 | cg12382864 | *PIK3AP1* | 10 | 0.0004 | 0.0057 | 2.72E-06 | 0.0049 |
| 243 | cg19646445 | *KCTD3* | 1 | -0.0004 | 0.0030 | 2.74E-06 | 0.0049 |
| 244 | cg05621091 | *ELP3* | 8 | -0.0001 | 0.0059 | 2.77E-06 | 0.0049 |
| 245 | cg26400169 | *FNIP1* | 5 | 0.0028 | 0.0051 | 2.77E-06 | 0.0049 |
| 246 | cg10761845 | *EEF2* | 19 | -0.0003 | 0.0056 | 2.82E-06 | 0.0050 |
| 247 | cg26680885 | *RNASET2* | 6 | -0.0002 | 0.0060 | 2.83E-06 | 0.0050 |
| 248 | cg17331254 | *PCMTD1* | 8 | -0.0003 | 0.0060 | 2.85E-06 | 0.0050 |
| 249 | cg10900643 | *C14orf80* | 14 | 0.0003 | 0.0017 | 2.85E-06 | 0.0050 |
| 250 | cg00133698 | *CAMK2D* | 4 | 0.0001 | 0.0032 | 2.87E-06 | 0.0050 |
| 251 | cg25053334 | *DDX47* | 12 | 0.0015 | 0.0061 | 2.88E-06 | 0.0050 |
| 252 | cg07469546 |  | 16 | 0.0009 | -0.0079 | 2.92E-06 | 0.0050 |
| 253 | cg07710481 | *SLITRK5* | 13 | 0.0047 | 0.0062 | 2.93E-06 | 0.0050 |
| 254 | cg04464559 | *PDSS2* | 6 | 0.0000 | 0.0034 | 2.96E-06 | 0.0050 |
| 255 | cg15773744 |  | 18 | -0.0012 | 0.0065 | 2.96E-06 | 0.0050 |
| 256 | cg19405229 | *LRRK1* | 15 | -0.0007 | -0.0073 | 2.97E-06 | 0.0050 |
| 257 | cg16713947 | *TAPBPL* | 12 | 0.0001 | 0.0066 | 2.98E-06 | 0.0050 |
| 258 | cg19324714 | *ASB18* | 2 | -0.0002 | 0.0053 | 3.01E-06 | 0.0051 |
| 259 | cg19741500 | *AHDC1* | 1 | 0.0000 | 0.0055 | 3.04E-06 | 0.0051 |
| 260 | cg26481784 | *ATPBD4* | 15 | 0.0003 | 0.0024 | 3.06E-06 | 0.0051 |
| 261 | cg14032204 | *ZNF575* | 19 | 0.0008 | 0.0052 | 3.14E-06 | 0.0052 |
| 262 | cg00791218 | *AMIGO1* | 1 | 0.0001 | 0.0028 | 3.20E-06 | 0.0053 |
| 263 | cg08070578 | *LMNB2* | 19 | 0.0010 | 0.0099 | 3.22E-06 | 0.0053 |
| 264 | cg16170181 | *GAB1* | 4 | -0.0004 | 0.0069 | 3.24E-06 | 0.0053 |
| 265 | cg00371920 | *RBMS1* | 2 | -0.0005 | 0.0065 | 3.26E-06 | 0.0053 |
| 266 | cg11278110 | *PLEKHM1P* | 17 | -0.0003 | 0.0061 | 3.40E-06 | 0.0055 |
| 267 | cg14232289 | *SSTR2* | 17 | 0.0003 | 0.0074 | 3.41E-06 | 0.0055 |
| 268 | cg27388983 | *ZNF256* | 19 | 0.0004 | 0.0023 | 3.43E-06 | 0.0055 |
| 269 | cg24673322 | *F5* | 1 | 0.0057 | 0.0028 | 3.46E-06 | 0.0056 |
| 270 | cg16552589 | *ASPM* | 1 | 0.0006 | 0.0070 | 3.47E-06 | 0.0056 |
| 271 | cg19411146 | *ESR1* | 6 | 0.0031 | 0.0055 | 3.51E-06 | 0.0056 |
| 272 | cg21528136 | *MRPL38* | 17 | -0.0002 | 0.0072 | 3.52E-06 | 0.0056 |
| 273 | cg19658926 | *CBX3* | 7 | 0.0003 | 0.0054 | 3.65E-06 | 0.0057 |
| 274 | cg12775935 | *RNF121* | 11 | 0.0027 | 0.0062 | 3.71E-06 | 0.0058 |
| 275 | cg09130556 | *CYP1B1* | 2 | 0.0040 | 0.0080 | 3.71E-06 | 0.0058 |
| 276 | cg24578679 | *CYP11A1* | 15 | 0.0002 | 0.0031 | 3.76E-06 | 0.0059 |
| 277 | cg19297845 | *MAPKBP1* | 15 | 0.0001 | 0.0053 | 3.84E-06 | 0.0059 |
| 278 | cg11065634 | *BCL11B* | 14 | 0.0000 | 0.0058 | 3.88E-06 | 0.0059 |
| 279 | cg11215207 |  | 11 | -0.0001 | 0.0088 | 3.88E-06 | 0.0059 |
| 280 | cg22987487 | *EBF2* | 8 | 0.0015 | 0.0050 | 3.88E-06 | 0.0059 |
| 281 | cg18178010 | *TNXB* | 6 | -0.0002 | 0.0067 | 3.89E-06 | 0.0059 |
| 282 | cg13958304 | *RSBN1* | 1 | 0.0012 | 0.0045 | 3.89E-06 | 0.0059 |
| 283 | cg06365094 | *HCFC1R1* | 16 | 0.0007 | 0.0064 | 3.94E-06 | 0.0060 |
| 284 | cg12475759 | *AJAP1* | 1 | 0.0030 | 0.0112 | 3.95E-06 | 0.0060 |
| 285 | cg24062754 | *RNF44* | 5 | -0.0002 | 0.0056 | 3.98E-06 | 0.0060 |
| 286 | cg16419498 | *MRPS16* | 10 | 0.0007 | 0.0039 | 4.08E-06 | 0.0062 |
| 287 | cg16734017 | *ARHGEF16* | 1 | 0.0002 | -0.0045 | 4.19E-06 | 0.0063 |
| 288 | cg05965745 | *PRDM16* | 1 | 0.0006 | 0.0099 | 4.25E-06 | 0.0064 |
| 289 | cg24139216 | *NDUFV1* | 11 | 0.0000 | 0.0050 | 4.26E-06 | 0.0064 |
| 290 | cg10166159 | *TRIM23* | 5 | 0.0005 | 0.0016 | 4.44E-06 | 0.0066 |
| 291 | cg23450324 | *KLHL23* | 2 | -0.0003 | 0.0068 | 4.47E-06 | 0.0066 |
| 292 | cg02682457 | *SYNE1* | 6 | 0.0001 | 0.0056 | 4.54E-06 | 0.0067 |
| 293 | cg06372962 | *ANKRD45* | 1 | 0.0012 | 0.0091 | 4.63E-06 | 0.0068 |
| 294 | cg22273015 | *RIC8A* | 11 | 0.0001 | 0.0062 | 4.63E-06 | 0.0068 |
| 295 | cg24638667 | *METTL2B* | 7 | 0.0011 | 0.0069 | 4.65E-06 | 0.0068 |
| 296 | cg21155461 | *ZNF544* | 19 | 0.0002 | 0.0026 | 4.66E-06 | 0.0068 |
| 297 | cg02150435 | *LOC374443* | 12 | 0.0003 | 0.0061 | 4.66E-06 | 0.0068 |
| 298 | cg26337277 | *TMPRSS2* | 21 | 0.0004 | 0.0037 | 4.72E-06 | 0.0068 |
| 299 | cg04588436 | *BCL11A* | 2 | -0.0047 | -0.0117 | 4.77E-06 | 0.0069 |
| 300 | cg06173626 | *ICOSLG* | 21 | 0.0003 | 0.0020 | 4.81E-06 | 0.0069 |
| 301 | cg21602557 | *EXT1* | 8 | 0.0022 | 0.0057 | 4.86E-06 | 0.0070 |
| 302 | cg08075060 | *BRP44L* | 6 | 0.0010 | 0.0024 | 4.86E-06 | 0.0070 |
| 303 | cg01071314 | *PRDM16* | 1 | 0.0002 | -0.0064 | 5.05E-06 | 0.0072 |
| 304 | cg10298701 | *CLDN6* | 16 | 0.0035 | 0.0040 | 5.19E-06 | 0.0074 |
| 305 | cg18456140 | *TCIRG1* | 11 | 0.0017 | -0.0086 | 5.21E-06 | 0.0074 |
| 306 | cg09479758 |  | 16 | -0.0006 | 0.0061 | 5.23E-06 | 0.0074 |
| 307 | cg08264694 | *SLC35E3* | 12 | 0.0001 | 0.0063 | 5.36E-06 | 0.0075 |
| 308 | cg21575078 | *C14orf80* | 14 | 0.0003 | 0.0017 | 5.37E-06 | 0.0075 |
| 309 | cg25339566 | *TCTEX1D1* | 1 | 0.0015 | 0.0065 | 5.46E-06 | 0.0077 |
| 310 | cg02584459 | *SLC16A12* | 10 | 0.0013 | 0.0089 | 5.48E-06 | 0.0077 |
| 311 | cg17759093 | *NAPB* | 20 | 0.0015 | 0.0057 | 5.74E-06 | 0.0080 |
| 312 | cg07666312 | *DDA1* | 19 | 0.0008 | 0.0060 | 6.14E-06 | 0.0085 |
| 313 | cg13951527 | *HIC1* | 17 | 0.0006 | 0.0092 | 6.20E-06 | 0.0086 |
| 314 | cg08710629 | *SLC7A5* | 16 | 0.0255 | 0.0161 | 6.24E-06 | 0.0086 |
| 315 | cg10170214 | *LZTS2* | 10 | 0.0023 | -0.0072 | 6.28E-06 | 0.0086 |
| 316 | cg26693760 | *RGL2* | 6 | 0.0292 | 0.0161 | 6.29E-06 | 0.0086 |
| 317 | cg01849007 | *XPO5* | 6 | 0.0006 | 0.0029 | 6.37E-06 | 0.0087 |
| 318 | cg20609493 |  | 15 | -0.0005 | 0.0041 | 6.43E-06 | 0.0087 |
| 319 | cg23892580 | *SMARCA4* | 19 | -0.0006 | 0.0058 | 6.52E-06 | 0.0088 |
| 320 | cg20390015 |  | 4 | -0.0068 | -0.0147 | 6.57E-06 | 0.0089 |
| 321 | cg21604320 | *MGST2* | 4 | 0.0011 | 0.0086 | 6.58E-06 | 0.0089 |
| 322 | cg18714679 | *RPL37* | 5 | 0.0009 | 0.0049 | 6.60E-06 | 0.0089 |
| 323 | cg19758033 | *DDOST* | 1 | -0.0016 | 0.0101 | 6.70E-06 | 0.0090 |
| 324 | cg27403822 | *TOR3A* | 1 | 0.0015 | 0.0024 | 6.71E-06 | 0.0090 |
| 325 | cg01714418 | *EDC3* | 15 | 0.0000 | 0.0035 | 6.72E-06 | 0.0090 |
| 326 | cg04845449 | *PEX26* | 22 | 0.0007 | 0.0068 | 6.74E-06 | 0.0090 |
| 327 | cg03519711 | *CYR61* | 1 | 0.0001 | 0.0052 | 6.92E-06 | 0.0092 |
| 328 | cg00583654 | *SLU7* | 5 | 0.0019 | 0.0062 | 6.97E-06 | 0.0092 |
| 329 | cg24482021 | *STX11* | 6 | -0.0014 | 0.0079 | 7.03E-06 | 0.0092 |
| 330 | cg02604407 | *SRI* | 7 | 0.0002 | 0.0049 | 7.04E-06 | 0.0092 |
| 331 | cg12405258 |  | 13 | 0.0004 | 0.0054 | 7.08E-06 | 0.0092 |
| 332 | cg06907160 | *YWHAG* | 7 | -0.0006 | 0.0058 | 7.09E-06 | 0.0092 |
| 333 | cg18503856 | *RGMB* | 5 | 0.0002 | 0.0038 | 7.12E-06 | 0.0093 |
| 334 | cg15561493 | *VWDE* | 7 | -0.0779 | -0.0625 | 7.23E-06 | 0.0094 |
| 335 | cg25626453 | *BAT2* | 6 | 0.0004 | -0.0077 | 7.25E-06 | 0.0094 |
| 336 | cg06910049 | *TMEM8A* | 16 | -0.0004 | -0.0072 | 7.28E-06 | 0.0094 |
| 337 | cg21612054 | *CCNDBP1* | 15 | -0.0007 | 0.0055 | 7.29E-06 | 0.0094 |
| 338 | cg18544826 | *TSHZ2* | 20 | -0.0003 | 0.0068 | 7.35E-06 | 0.0094 |
| 339 | cg00920990 | *NQO2* | 6 | 0.0017 | 0.0028 | 7.36E-06 | 0.0094 |
| 340 | cg17331461 | *ATXN7L1* | 7 | -0.0007 | 0.0060 | 7.43E-06 | 0.0095 |
| 341 | cg15834388 | *NECAB1* | 8 | 0.0191 | 0.0123 | 7.52E-06 | 0.0096 |
| 342 | cg15501281 | *SLC24A3* | 20 | -0.0003 | 0.0065 | 7.59E-06 | 0.0096 |
| 343 | cg11832804 | *TERT* | 5 | -0.0028 | -0.0061 | 7.77E-06 | 0.0098 |
| 344 | cg22237200 | *ATP1B2* | 17 | -0.0010 | 0.0034 | 7.83E-06 | 0.0099 |
| 345 | cg16089443 | *LIN52* | 14 | 0.0011 | 0.0058 | 7.92E-06 | 0.0100 |
| 346 | cg09701102 | *NDUFV1* | 11 | -0.0101 | -0.0160 | 7.93E-06 | 0.0100 |
| 347 | cg16112844 | *PHLDA2* | 11 | 0.0000 | 0.0058 | 8.04E-06 | 0.0101 |
| 348 | cg25385272 | *TAF1A* | 1 | 0.0017 | 0.0024 | 8.06E-06 | 0.0101 |
| 349 | cg01727854 | *CMIP* | 16 | -0.0057 | -0.0106 | 8.10E-06 | 0.0101 |
| 350 | cg21836627 |  | 6 | -0.0002 | 0.0065 | 8.28E-06 | 0.0102 |
| 351 | cg25786436 | *PRKAG2* | 7 | 0.0004 | 0.0031 | 8.50E-06 | 0.0105 |
| 352 | cg10377270 | *ZBTB22* | 6 | 0.0001 | 0.0038 | 8.55E-06 | 0.0105 |
| 353 | cg05038804 | *GSK3B* | 3 | 0.0006 | 0.0037 | 8.69E-06 | 0.0107 |
| 354 | cg19651132 | *KCNA1* | 12 | 0.0002 | 0.0012 | 8.82E-06 | 0.0108 |
| 355 | cg12835924 | *ZNF239* | 10 | -0.0002 | 0.0052 | 8.89E-06 | 0.0108 |
| 356 | cg26135345 |  | 6 | 0.0012 | 0.0038 | 9.49E-06 | 0.0116 |
| 357 | cg02736548 | *FAM109B* | 22 | 0.0001 | 0.0038 | 9.55E-06 | 0.0116 |
| 358 | cg19201719 | *DOCK8* | 9 | -0.0001 | 0.0060 | 9.62E-06 | 0.0116 |
| 359 | cg08298555 | *CACNA1H* | 16 | -0.0022 | -0.0136 | 9.63E-06 | 0.0116 |
| 360 | cg03734402 | *RNF207* | 1 | 0.0013 | 0.0059 | 9.66E-06 | 0.0116 |
| 361 | cg05410102 | *CX3CL1* | 16 | 0.0006 | -0.0069 | 9.66E-06 | 0.0116 |
| 362 | cg10158093 | *DPF3* | 14 | 0.0009 | 0.0070 | 1.00E-05 | 0.0120 |
| 363 | cg13576505 |  | 4 | 0.0025 | -0.0079 | 1.05E-05 | 0.0125 |
| 364 | cg25626175 | *CXADR* | 21 | -0.0001 | 0.0060 | 1.06E-05 | 0.0126 |
| 365 | cg09389160 | *TUBB* | 6 | 0.0055 | 0.0133 | 1.07E-05 | 0.0127 |
| 366 | cg04714201 | *ABCF1* | 6 | 0.0006 | 0.0041 | 1.08E-05 | 0.0127 |
| 367 | cg23649435 | *STOX2* | 4 | 0.0007 | 0.0034 | 1.11E-05 | 0.0131 |
| 368 | cg07175090 |  | 1 | -0.0011 | -0.0100 | 1.12E-05 | 0.0132 |
| 369 | cg21035471 | *CANT1* | 17 | 0.0007 | 0.0063 | 1.12E-05 | 0.0132 |
| 370 | cg25679431 | *GPD1L* | 3 | -0.0001 | -0.0145 | 1.13E-05 | 0.0132 |
| 371 | cg02300308 | *EGFL8* | 6 | -0.0149 | -0.0229 | 1.14E-05 | 0.0133 |
| 372 | cg07953400 | *PIGQ* | 16 | -0.0004 | 0.0077 | 1.15E-05 | 0.0133 |
| 373 | cg08708599 |  | 1 | 0.0012 | 0.0040 | 1.17E-05 | 0.0135 |
| 374 | cg18442587 | *VASH1* | 14 | 0.0036 | 0.0092 | 1.17E-05 | 0.0135 |
| 375 | cg15538669 | *HSPA9* | 5 | -0.0002 | 0.0043 | 1.18E-05 | 0.0135 |
| 376 | cg26614578 | *C12orf52* | 12 | 0.0027 | 0.0062 | 1.18E-05 | 0.0135 |
| 377 | cg16307498 | *PA2G4* | 12 | -0.0004 | 0.0056 | 1.18E-05 | 0.0135 |
| 378 | cg15596816 | *LOC100272146* | 17 | 0.0001 | 0.0025 | 1.20E-05 | 0.0136 |
| 379 | cg16014122 | *TREX1* | 3 | -0.0050 | -0.0115 | 1.20E-05 | 0.0136 |
| 380 | cg12753558 | *ZNF496* | 1 | -0.0074 | -0.0093 | 1.21E-05 | 0.0138 |
| 381 | cg12721546 | *BCLAF1* | 6 | 0.0005 | 0.0036 | 1.25E-05 | 0.0142 |
| 382 | cg20898347 | *TMEM11* | 17 | 0.0004 | 0.0052 | 1.27E-05 | 0.0144 |
| 383 | cg13722539 | *PRDX5* | 11 | 0.0001 | 0.0016 | 1.29E-05 | 0.0145 |
| 384 | cg19430577 | *STEAP4* | 7 | 0.0003 | 0.0023 | 1.32E-05 | 0.0149 |
| 385 | cg01124027 | *STX18* | 4 | 0.0000 | 0.0012 | 1.33E-05 | 0.0149 |
| 386 | cg05600350 |  | 4 | 0.0003 | 0.0064 | 1.33E-05 | 0.0149 |
| 387 | cg00886954 | *PLEC1* | 8 | 0.0007 | 0.0019 | 1.34E-05 | 0.0149 |
| 388 | cg21968324 | *CCDC85A* | 2 | 0.0004 | 0.0011 | 1.36E-05 | 0.0151 |
| 389 | cg18174326 | *PASK* | 2 | 0.0000 | 0.0011 | 1.36E-05 | 0.0151 |
| 390 | cg22627353 | *CHST8* | 19 | -0.0061 | -0.0096 | 1.37E-05 | 0.0152 |
| 391 | cg14047494 | *C9orf46* | 9 | 0.0000 | -0.0072 | 1.37E-05 | 0.0152 |
| 392 | cg11452292 | *ATP5L* | 11 | 0.0010 | 0.0061 | 1.39E-05 | 0.0153 |
| 393 | cg08195943 | *TRH* | 3 | -0.0002 | 0.0028 | 1.39E-05 | 0.0153 |
| 394 | cg23779240 |  | 10 | 0.0241 | 0.0213 | 1.43E-05 | 0.0156 |
| 395 | cg02619867 |  | 1 | -0.0045 | -0.0130 | 1.43E-05 | 0.0156 |
| 396 | cg27208903 | *SLC7A5P1* | 16 | 0.0137 | 0.0075 | 1.43E-05 | 0.0156 |
| 397 | cg22661129 | *UNC5D* | 8 | -0.0031 | -0.0107 | 1.43E-05 | 0.0156 |
| 398 | cg26379672 | *ITPR1* | 3 | 0.0054 | 0.0034 | 1.44E-05 | 0.0157 |
| 399 | cg15065561 |  | 19 | -0.0046 | -0.0189 | 1.45E-05 | 0.0157 |
| 400 | cg01857594 | *ZNF540* | 19 | -0.0003 | 0.0016 | 1.45E-05 | 0.0157 |
| 401 | cg16297011 |  | 4 | 0.0011 | 0.0016 | 1.46E-05 | 0.0157 |
| 402 | cg14787260 | *STAT5B* | 17 | -0.0001 | 0.0053 | 1.46E-05 | 0.0157 |
| 403 | cg22802994 | *KIAA1543* | 19 | 0.0006 | -0.0240 | 1.51E-05 | 0.0162 |
| 404 | cg03973705 | *PRKCB* | 16 | -0.0042 | -0.0092 | 1.53E-05 | 0.0163 |
| 405 | cg03979206 | *HSPA1B* | 6 | 0.0007 | 0.0038 | 1.55E-05 | 0.0166 |
| 406 | cg06478814 | *C16orf80* | 16 | 0.0001 | 0.0039 | 1.56E-05 | 0.0166 |
| 407 | cg06293395 |  | 16 | -0.0018 | -0.0053 | 1.56E-05 | 0.0166 |
| 408 | cg25542907 | *SFRS7* | 2 | 0.0028 | 0.0105 | 1.57E-05 | 0.0166 |
| 409 | cg13159120 |  | 17 | -0.0038 | -0.0053 | 1.59E-05 | 0.0168 |
| 410 | cg03732087 | *AGBL4* | 1 | -0.0021 | 0.0096 | 1.63E-05 | 0.0172 |
| 411 | cg19225178 |  | 12 | -0.0019 | -0.0118 | 1.65E-05 | 0.0174 |
| 412 | cg21244086 | *SHF* | 15 | -0.0027 | 0.0160 | 1.69E-05 | 0.0178 |
| 413 | cg17867823 | *PRIC285* | 20 | -0.0007 | -0.0067 | 1.70E-05 | 0.0178 |
| 414 | cg16622514 | *COL4A3BP* | 5 | -0.0078 | -0.0114 | 1.71E-05 | 0.0178 |
| 415 | cg14142965 | *TXNRD1* | 12 | 0.0002 | 0.0056 | 1.76E-05 | 0.0183 |
| 416 | cg08651378 | *C19orf25* | 19 | 0.0001 | 0.0023 | 1.78E-05 | 0.0185 |
| 417 | cg08520228 | *ZNF225* | 19 | 0.0032 | 0.0059 | 1.85E-05 | 0.0191 |
| 418 | cg05005113 | *NANP* | 20 | 0.0003 | 0.0023 | 1.86E-05 | 0.0192 |
| 419 | cg05961809 | *SFRP2* | 4 | 0.0014 | 0.0066 | 1.87E-05 | 0.0193 |
| 420 | cg00489213 | *FAM120B* | 6 | -0.0037 | -0.0137 | 1.90E-05 | 0.0194 |
| 421 | cg25137030 | *C15orf48* | 15 | -0.0001 | 0.0008 | 1.91E-05 | 0.0195 |
| 422 | cg14345980 |  | 14 | 0.0003 | 0.0025 | 1.92E-05 | 0.0195 |
| 423 | cg19896639 | *SLC25A33* | 1 | 0.0034 | 0.0049 | 1.92E-05 | 0.0195 |
| 424 | cg03405515 | *MAP9* | 4 | 0.0002 | 0.0012 | 1.92E-05 | 0.0195 |
| 425 | cg17948627 | *PSMB2* | 1 | 0.0014 | 0.0040 | 1.94E-05 | 0.0197 |
| 426 | cg07944863 | *RGS7* | 1 | 0.0002 | 0.0108 | 2.01E-05 | 0.0203 |
| 427 | ch.19.36684304F |  | 19 | 0.0001 | 0.0036 | 2.02E-05 | 0.0203 |
| 428 | cg22782017 |  | 6 | -0.0086 | -0.0115 | 2.02E-05 | 0.0203 |
| 429 | cg17357132 | *C11orf1* | 11 | 0.0011 | 0.0024 | 2.05E-05 | 0.0205 |
| 430 | cg01460814 |  | 16 | 0.0023 | -0.0084 | 2.08E-05 | 0.0209 |
| 431 | cg18567174 | *LBXCOR1* | 15 | -0.0003 | 0.0031 | 2.11E-05 | 0.0210 |
| 432 | cg07173972 | *SLC17A9* | 20 | 0.0120 | 0.0166 | 2.12E-05 | 0.0211 |
| 433 | cg10068403 |  | 14 | -0.0041 | -0.0111 | 2.13E-05 | 0.0211 |
| 434 | cg02595832 | *FOXI2* | 10 | 0.0053 | 0.0030 | 2.17E-05 | 0.0215 |
| 435 | cg17558973 | *C5orf42* | 5 | 0.0002 | 0.0069 | 2.25E-05 | 0.0222 |
| 436 | cg00488788 | *ONECUT1* | 15 | 0.0018 | 0.0073 | 2.35E-05 | 0.0232 |
| 437 | cg17491622 | *VWA1* | 1 | 0.0110 | 0.0045 | 2.39E-05 | 0.0235 |
| 438 | cg19824907 | *GPC6* | 13 | 0.0011 | 0.0065 | 2.42E-05 | 0.0237 |
| 439 | cg05092988 | *DSCAML1* | 11 | -0.0031 | -0.0089 | 2.45E-05 | 0.0240 |
| 440 | cg06018203 | *ZNF639* | 3 | 0.0004 | 0.0046 | 2.48E-05 | 0.0242 |
| 441 | cg19472078 | *C1QTNF1* | 17 | 0.0012 | 0.0054 | 2.49E-05 | 0.0243 |
| 442 | cg00083535 | *TRAPPC9* | 8 | -0.0020 | -0.0150 | 2.49E-05 | 0.0243 |
| 443 | cg06610204 | *DHRS7B* | 17 | -0.0004 | 0.0058 | 2.51E-05 | 0.0244 |
| 444 | cg16841327 |  | 22 | -0.0011 | 0.0027 | 2.52E-05 | 0.0244 |
| 445 | cg00800773 | *PRKCD* | 3 | 0.0013 | 0.0079 | 2.52E-05 | 0.0244 |
| 446 | cg24029517 | *HS3ST1* | 4 | 0.0116 | 0.0105 | 2.59E-05 | 0.0250 |
| 447 | cg08614097 | *OR5B21* | 11 | -0.0043 | -0.0106 | 2.63E-05 | 0.0253 |
| 448 | cg01666550 | *LRP5* | 11 | -0.0034 | -0.0066 | 2.66E-05 | 0.0255 |
| 449 | cg24032214 | *CNOT3* | 19 | -0.0043 | -0.0097 | 2.67E-05 | 0.0256 |
| 450 | cg23143978 | *RBM33* | 7 | 0.0009 | 0.0091 | 2.71E-05 | 0.0259 |
| 451 | cg01780171 |  | 2 | -0.0013 | -0.0185 | 2.71E-05 | 0.0259 |
| 452 | cg13679776 | *CACHD1* | 1 | 0.0025 | 0.0105 | 2.82E-05 | 0.0268 |
| 453 | cg19592502 | *SEMA3C* | 7 | -0.0121 | -0.0094 | 2.82E-05 | 0.0268 |
| 454 | cg14565265 | *LRRC8D* | 1 | 0.0003 | 0.0019 | 2.85E-05 | 0.0270 |
| 455 | cg07964833 | *FCAR* | 19 | -0.0016 | 0.0053 | 2.94E-05 | 0.0278 |
| 456 | cg09829263 | *RCBTB2* | 13 | 0.0011 | 0.0044 | 2.95E-05 | 0.0278 |
| 457 | cg18084791 | *SLAMF8* | 1 | -0.0032 | -0.0148 | 2.98E-05 | 0.0280 |
| 458 | cg09049376 | *OLA1* | 2 | 0.0003 | 0.0011 | 3.01E-05 | 0.0282 |
| 459 | cg18052984 | *ATF1* | 12 | -0.0006 | 0.0086 | 3.02E-05 | 0.0283 |
| 460 | cg13252204 | *TTC4* | 1 | 0.0034 | 0.0021 | 3.06E-05 | 0.0285 |
| 461 | cg18052665 | *DPPA5* | 6 | -0.0154 | -0.0173 | 3.10E-05 | 0.0288 |
| 462 | cg15558759 | *PHLDB1* | 11 | 0.0001 | 0.0040 | 3.10E-05 | 0.0288 |
| 463 | cg16045355 | *MYST4* | 10 | -0.0039 | -0.0094 | 3.11E-05 | 0.0288 |
| 464 | cg24093411 | *TCF7* | 5 | 0.0042 | 0.0041 | 3.13E-05 | 0.0289 |
| 465 | cg15335436 |  | 5 | 0.0124 | 0.0143 | 3.14E-05 | 0.0289 |
| 466 | cg00549973 |  | 6 | -0.0134 | -0.0173 | 3.15E-05 | 0.0290 |
| 467 | cg22466043 | *CDC10L* | 10 | -0.0006 | 0.0026 | 3.16E-05 | 0.0290 |
| 468 | cg05258294 | *ZNF598* | 16 | 0.0011 | -0.0075 | 3.16E-05 | 0.0290 |
| 469 | cg15707332 | *CHMP4B* | 20 | -0.0058 | -0.0079 | 3.22E-05 | 0.0294 |
| 470 | cg19314470 | *RBPMS* | 8 | -0.0132 | -0.0161 | 3.26E-05 | 0.0298 |
| 471 | cg12002303 |  | 15 | 0.0039 | 0.0069 | 3.33E-05 | 0.0303 |
| 472 | cg05682094 | *PCNA* | 20 | 0.0007 | 0.0013 | 3.35E-05 | 0.0305 |
| 473 | cg04840739 | *ZXDC* | 3 | 0.0043 | 0.0107 | 3.45E-05 | 0.0313 |
| 474 | cg19729366 |  | 1 | -0.0097 | -0.0085 | 3.48E-05 | 0.0316 |
| 475 | cg19315466 | *KIF26B* | 1 | -0.0026 | -0.0094 | 3.49E-05 | 0.0316 |
| 476 | cg18270113 |  | 7 | -0.0018 | 0.0133 | 3.54E-05 | 0.0320 |
| 477 | cg05702505 | *STRBP* | 9 | 0.0046 | 0.0102 | 3.60E-05 | 0.0325 |
| 478 | cg02633148 | *GABRA2* | 4 | 0.0064 | 0.0078 | 3.62E-05 | 0.0325 |
| 479 | cg02448207 | *BBS9* | 7 | 0.0133 | 0.0105 | 3.62E-05 | 0.0325 |
| 480 | cg09362543 | *PODN* | 1 | 0.0020 | -0.0099 | 3.62E-05 | 0.0325 |
| 481 | cg01069804 | *RPS27L* | 15 | 0.0005 | 0.0040 | 3.65E-05 | 0.0326 |
| 482 | cg03383941 | *WFDC2* | 20 | 0.0013 | 0.0047 | 3.66E-05 | 0.0327 |
| 483 | cg22028544 | *C8orf59* | 8 | -0.0012 | 0.0054 | 3.68E-05 | 0.0327 |
| 484 | cg19132762 | *EPM2AIP1* | 3 | -0.0012 | 0.0079 | 3.68E-05 | 0.0327 |
| 485 | cg18085998 | *ZSCAN20* | 1 | -0.0004 | 0.0053 | 3.69E-05 | 0.0327 |
| 486 | cg23545872 | *KCNMA1* | 10 | -0.0151 | -0.0087 | 3.69E-05 | 0.0327 |
| 487 | cg15480630 | *CORO2B* | 15 | -0.0011 | -0.0105 | 3.74E-05 | 0.0330 |
| 488 | cg21606780 | *LHX9* | 1 | 0.0057 | 0.0065 | 3.74E-05 | 0.0330 |
| 489 | cg24466448 | *SLC43A2* | 17 | 0.0024 | -0.0088 | 3.74E-05 | 0.0330 |
| 490 | cg26690949 |  | 20 | 0.0031 | 0.0064 | 3.92E-05 | 0.0344 |
| 491 | cg16915821 | *DKK3* | 11 | -0.0006 | 0.0038 | 3.96E-05 | 0.0347 |
| 492 | cg02858826 | *RPS6KL1* | 14 | -0.0052 | -0.0087 | 4.01E-05 | 0.0351 |
| 493 | cg09728607 |  | 16 | 0.0005 | 0.0012 | 4.02E-05 | 0.0351 |
| 494 | cg10024437 |  | 14 | -0.0004 | 0.0026 | 4.06E-05 | 0.0354 |
| 495 | cg27182527 | *WIPF3* | 7 | -0.0085 | -0.0127 | 4.10E-05 | 0.0356 |
| 496 | cg19515446 | *HIST1H1T* | 6 | -0.0082 | -0.0155 | 4.11E-05 | 0.0356 |
| 497 | cg18673954 | *INA* | 10 | -0.0009 | 0.0025 | 4.13E-05 | 0.0357 |
| 498 | cg17001101 |  | 1 | -0.0018 | 0.0065 | 4.16E-05 | 0.0359 |
| 499 | cg18646207 | *VAX1* | 10 | 0.0005 | 0.0069 | 4.18E-05 | 0.0360 |
| 500 | cg00611535 | *RAB5B* | 12 | 0.0016 | 0.0040 | 4.25E-05 | 0.0365 |
| 501 | cg21948071 | *FRMD4B* | 3 | -0.0006 | -0.0097 | 4.26E-05 | 0.0366 |
| 502 | cg17169123 | *CD63* | 12 | 0.0007 | 0.0035 | 4.38E-05 | 0.0376 |
| 503 | cg10570241 |  | 7 | 0.0164 | 0.0097 | 4.41E-05 | 0.0377 |
| 504 | cg02642549 | *LHFPL2* | 5 | 0.0002 | 0.0073 | 4.41E-05 | 0.0377 |
| 505 | cg20517154 | *MTUS1* | 8 | 0.0002 | 0.0009 | 4.44E-05 | 0.0378 |
| 506 | cg11616651 | *AMH* | 19 | 0.0038 | 0.0049 | 4.45E-05 | 0.0379 |
| 507 | cg02850468 | *NPSR1* | 7 | -0.0302 | -0.0174 | 4.47E-05 | 0.0379 |
| 508 | cg05647756 | *UNK* | 17 | -0.0001 | -0.0065 | 4.47E-05 | 0.0379 |
| 509 | cg16177875 | *CEP152* | 15 | -0.0001 | 0.0035 | 4.48E-05 | 0.0379 |
| 510 | cg21935449 | *KIAA1310* | 2 | -0.0029 | -0.0105 | 4.56E-05 | 0.0385 |
| 511 | cg09013480 |  | 12 | -0.0018 | -0.0206 | 4.58E-05 | 0.0385 |
| 512 | cg01530634 | *LOC100289511* | 14 | 0.0009 | 0.0037 | 4.60E-05 | 0.0386 |
| 513 | cg22687807 | *LYNX1* | 8 | 0.0002 | 0.0020 | 4.61E-05 | 0.0386 |
| 514 | cg01468505 | *ORC2L* | 2 | 0.0017 | 0.0034 | 4.63E-05 | 0.0388 |
| 515 | cg11230083 | *ATP11A* | 13 | -0.0152 | -0.0185 | 4.79E-05 | 0.0401 |
| 516 | cg13596049 | *FAM59A* | 18 | 0.0013 | 0.0022 | 4.84E-05 | 0.0404 |
| 517 | cg10316635 | *INADL* | 1 | 0.0085 | 0.0102 | 4.88E-05 | 0.0407 |
| 518 | cg05891181 | *HCG27* | 6 | 0.0015 | 0.0046 | 4.98E-05 | 0.0413 |
| 519 | cg04652903 | *WDR27* | 6 | -0.0172 | -0.0177 | 4.98E-05 | 0.0413 |
| 520 | cg11949606 | *SYNJ1* | 21 | 0.0039 | -0.0144 | 5.03E-05 | 0.0417 |
| 521 | cg23716101 | *POLE2* | 14 | 0.0018 | 0.0040 | 5.05E-05 | 0.0418 |
| 522 | cg25078150 |  | 10 | 0.0013 | 0.0048 | 5.11E-05 | 0.0420 |
| 523 | cg16659246 | *LCORL* | 4 | 0.0009 | 0.0009 | 5.14E-05 | 0.0421 |
| 524 | cg00971834 | *COL13A1* | 10 | 0.0001 | 0.0009 | 5.20E-05 | 0.0424 |
| 525 | cg14493920 |  | 9 | 0.0002 | 0.0045 | 5.20E-05 | 0.0424 |
| 526 | cg24345247 | *HIST1H4A* | 6 | 0.0030 | 0.0065 | 5.27E-05 | 0.0429 |
| 527 | cg14047844 | *FLJ42709* | 5 | 0.0037 | 0.0046 | 5.27E-05 | 0.0429 |
| 528 | cg01370181 | *NR2F2* | 15 | 0.0010 | 0.0010 | 5.34E-05 | 0.0434 |
| 529 | cg02630129 | *NRIP1* | 21 | 0.0006 | 0.0041 | 5.52E-05 | 0.0448 |
| 530 | cg26038318 | *SPAG4* | 20 | -0.0011 | 0.0124 | 5.56E-05 | 0.0450 |
| 531 | cg27287791 | *CEP152* | 15 | -0.0005 | 0.0051 | 5.58E-05 | 0.0451 |
| 532 | cg09490387 | *GFER* | 16 | -0.0008 | 0.0089 | 5.60E-05 | 0.0451 |
| 533 | cg27128984 |  | 5 | -0.0006 | 0.0034 | 5.61E-05 | 0.0451 |
| 534 | cg22134370 |  | 5 | -0.0021 | 0.0134 | 5.66E-05 | 0.0454 |
| 535 | cg15674193 | *LRRFIP1* | 2 | 0.0009 | 0.0037 | 5.69E-05 | 0.0456 |
| 536 | cg08012045 | *A2M* | 12 | -0.0067 | -0.0101 | 5.74E-05 | 0.0459 |
| 537 | cg21035402 | *LRFN3* | 19 | -0.0024 | -0.0105 | 5.75E-05 | 0.0459 |
| 538 | cg23741863 | *PGCP* | 8 | 0.0021 | 0.0042 | 5.76E-05 | 0.0459 |
| 539 | cg10131465 |  | 11 | -0.0077 | -0.0122 | 5.85E-05 | 0.0465 |
| 540 | cg03451296 | *CRIP2* | 14 | 0.0000 | 0.0014 | 5.94E-05 | 0.0472 |
| 541 | cg05607246 | *CITED2* | 6 | -0.0002 | 0.0025 | 5.98E-05 | 0.0474 |
| 542 | cg23223091 | *TNXB* | 6 | -0.0046 | -0.0090 | 5.99E-05 | 0.0474 |
| 543 | cg05097306 |  | 10 | 0.0012 | 0.0123 | 6.00E-05 | 0.0474 |
| 544 | cg16807089 | *FAM115A* | 7 | 0.0045 | 0.0122 | 6.03E-05 | 0.0475 |
| 545 | cg05354904 | *CBFA2T3* | 16 | -0.0054 | -0.0184 | 6.06E-05 | 0.0477 |
| 546 | cg13755796 |  | 4 | 0.0000 | 0.0017 | 6.09E-05 | 0.0479 |
| 547 | cg02317400 | *VENTX* | 10 | 0.0018 | 0.0139 | 6.12E-05 | 0.0480 |
| 548 | cg21068911 | *ELMOD1* | 11 | 0.0012 | 0.0012 | 6.19E-05 | 0.0485 |
| 549 | cg08075308 | *TTC27* | 2 | -0.0001 | 0.0028 | 6.31E-05 | 0.0494 |
| 550 | cg13847963 | *IL28RA* | 1 | -0.0004 | 0.0019 | 6.35E-05 | 0.0495 |
| 551 | cg17752846 | *MTX2* | 2 | -0.0002 | 0.0029 | 6.38E-05 | 0.0497 |
| 552 | cg10658516 | *TMEM131* | 2 | 0.0031 | 0.0115 | 6.42E-05 | 0.0499 |
|  |  |  |  |  |  |  |  |
