# Supplementary material for: Prenatal cannabis exposure is associated with alterations in offspring DNA methylation at genes involved in neurodevelopment, across the life course
Source: Mol Psychiatry. 2024 Sep 14;30(4):1418–29. doi: 10.1038/s41380-024-02752-w (PMC11919715; doi:10.1038/s41380-024-02752-w)
Supplement: Supplementary file 5 — Supplementary Table 4 [file 41380_2024_2752_MOESM5_ESM.docx]

**Supplementary Table 4**

The top 20 differentially methylated CpG sites in response to PTE at ~27 y (CHDS)

| **Rank** | **IlmnID** | **Gene Name** | **CHR** | **Beta difference** | **logFC** | **P.Value** | **adj.P.Val** |
| --- | --- | --- | --- | --- | --- | --- | --- |
| 1 | cg15507334 | *FRMD4A* | 10 | 0.0809 | 0.0839 | 1.59E-09 | 0.0011 |
| 2 | cg25464840 | *FRMD4A* | 10 | 0.0607 | 0.0631 | 4.71E-08 | 0.0165 |
| 3 | cg11813497 | *FRMD4A* | 10 | 0.0666 | 0.0694 | 6.13E-07 | 0.1430 |
| 4 | cg15766464 |  | 5 | 0.0469 | 0.0609 | 7.71E-06 | 1.0000 |
| 5 | cg27339941 |  | 9 | 0.0550 | 0.0608 | 1.11E-05 | 1.0000 |
| 6 | cg24601030 | *DIXDC1* | 11 | 0.0547 | 0.0636 | 1.13E-05 | 1.0000 |
| 7 | cg19590598 |  | 2 | 0.0525 | 0.0865 | 1.38E-05 | 1.0000 |
| 8 | cg14630801 | *FRMD4A* | 10 | 0.0684 | 0.0689 | 1.77E-05 | 1.0000 |
| 9 | cg17230098 | *PHF12* | 17 | -0.1113 | -0.1480 | 1.81E-05 | 1.0000 |
| 10 | cg17949821 | *GAS7* | 17 | 0.0264 | 0.0296 | 1.82E-05 | 1.0000 |
| 11 | cg03287658 |  | 6 | 0.0722 | 0.1035 | 1.93E-05 | 1.0000 |
| 12 | cg25822369 |  | 2 | 0.1058 | 0.1581 | 2.56E-05 | 1.0000 |
| 13 | cg08373610 | *RFTN2* | 2 | 0.0706 | 0.0822 | 2.58E-05 | 1.0000 |
| 14 | cg24369561 | *MIPOL1* | 14 | 0.1469 | 0.1911 | 2.80E-05 | 1.0000 |
| 15 | cg21314058 | *PRIMA1* | 14 | 0.0567 | 0.0597 | 3.56E-05 | 1.0000 |
| 16 | cg03240518 | *DIXDC1* | 11 | 0.0217 | 0.0367 | 4.00E-05 | 1.0000 |
| 17 | cg02858514 | *EPB41L3* | 18 | 0.0377 | 0.0512 | 4.01E-05 | 1.0000 |
| 18 | cg06671242 | *PRSS23* | 11 | 0.0403 | 0.0383 | 4.27E-05 | 1.0000 |
| 19 | cg09059880 | *C11orf87* | 11 | 0.0489 | 0.0510 | 4.75E-05 | 1.0000 |
| 20 | cg13659405 |  | 2 | 0.0409 | 0.0644 | 5.50E-05 | 1.0000 |
