# Supplementary figures and images for: Prenatal cannabis exposure is associated with alterations in offspring DNA methylation at genes involved in neurodevelopment, across the life course
Source: Mol Psychiatry. 2024 Sep 14;30(4):1418–29. doi: 10.1038/s41380-024-02752-w (PMC11919715; doi:10.1038/s41380-024-02752-w)

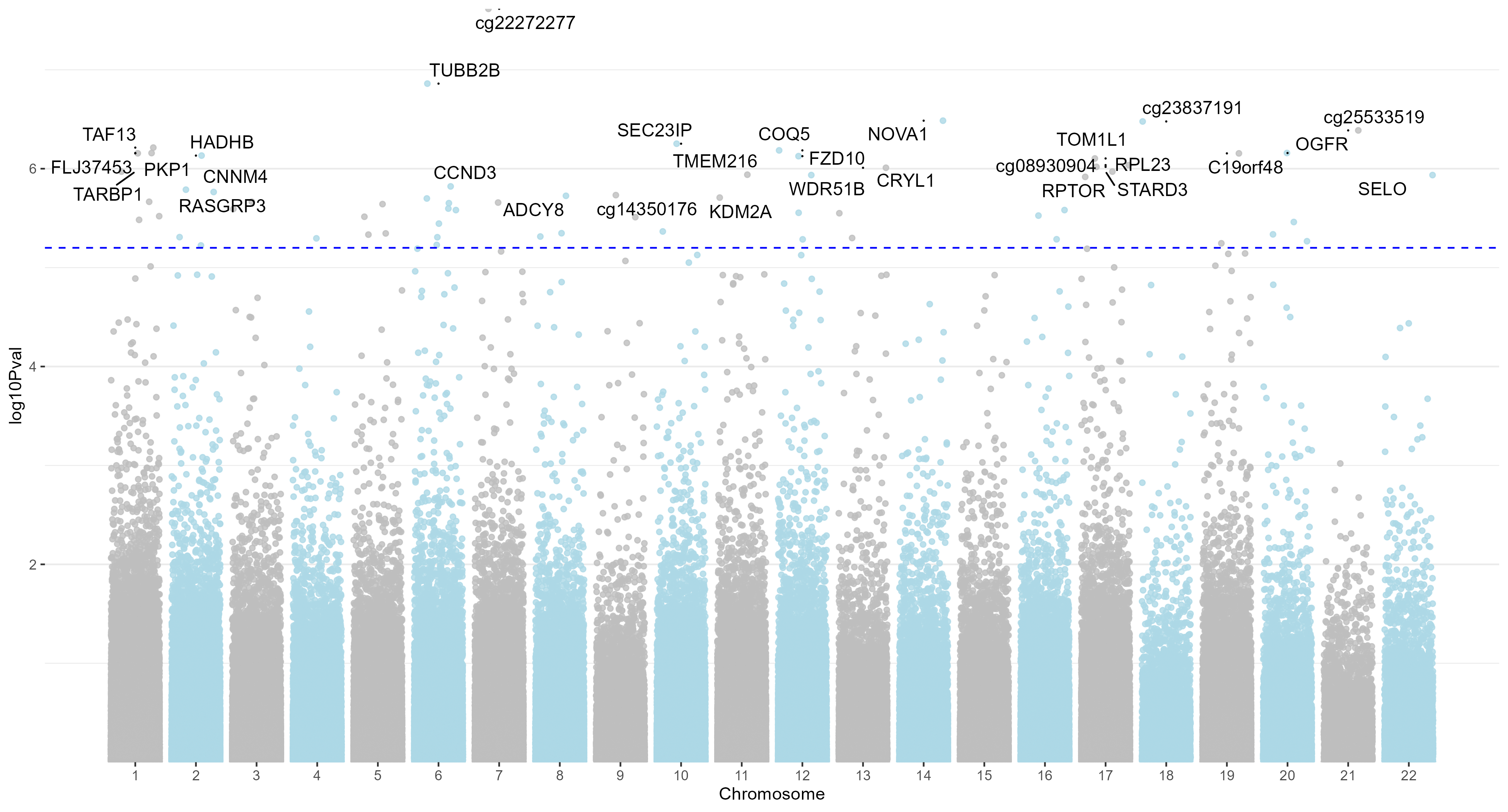

Supplement: Supplementary file 6 — Supplementary Fig. 1 [file 41380_2024_2752_MOESM6_ESM.tif]

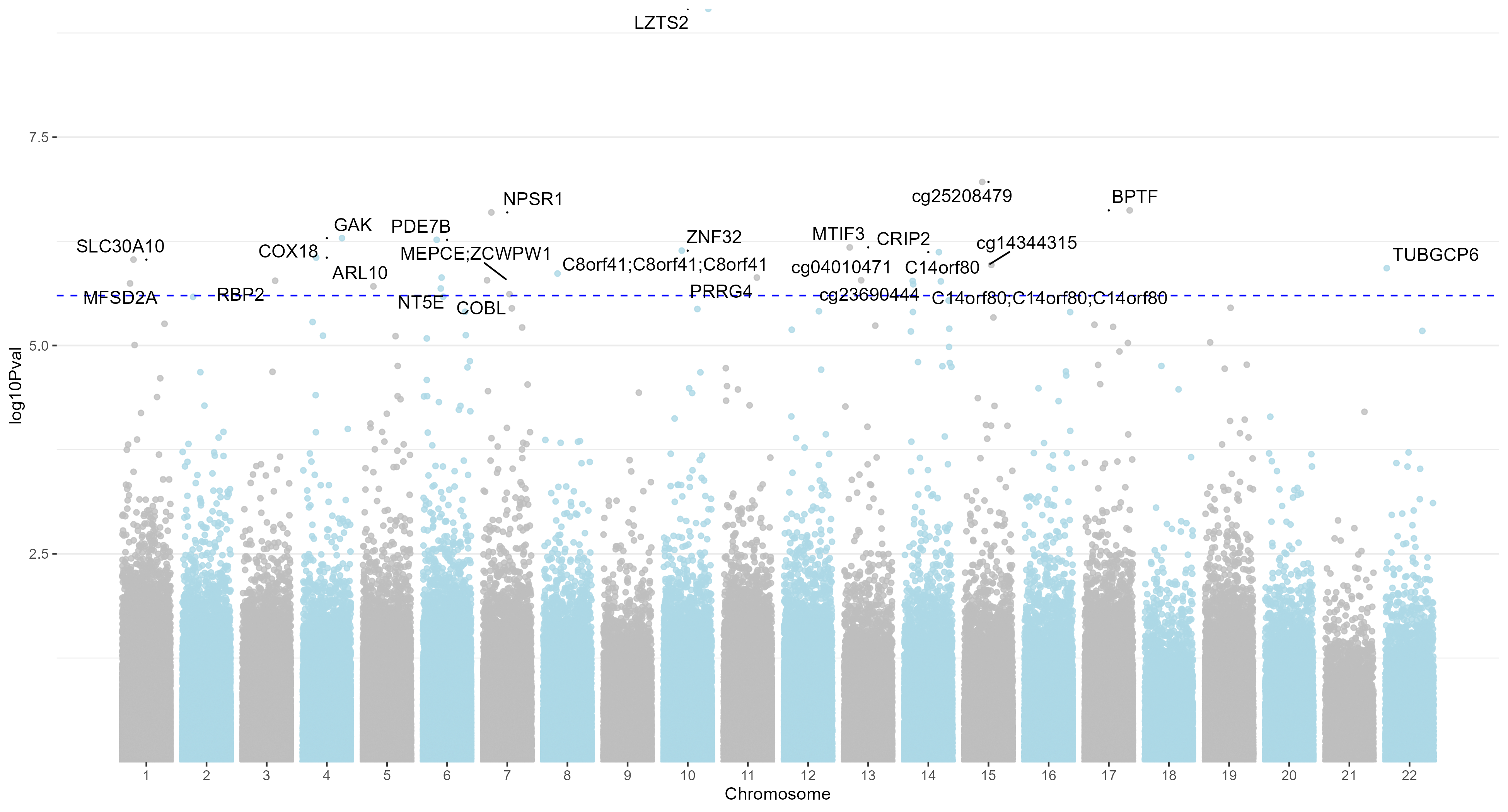

Supplement: Supplementary file 7 — Supplementary Fig. 2 [file 41380_2024_2752_MOESM7_ESM.tif]

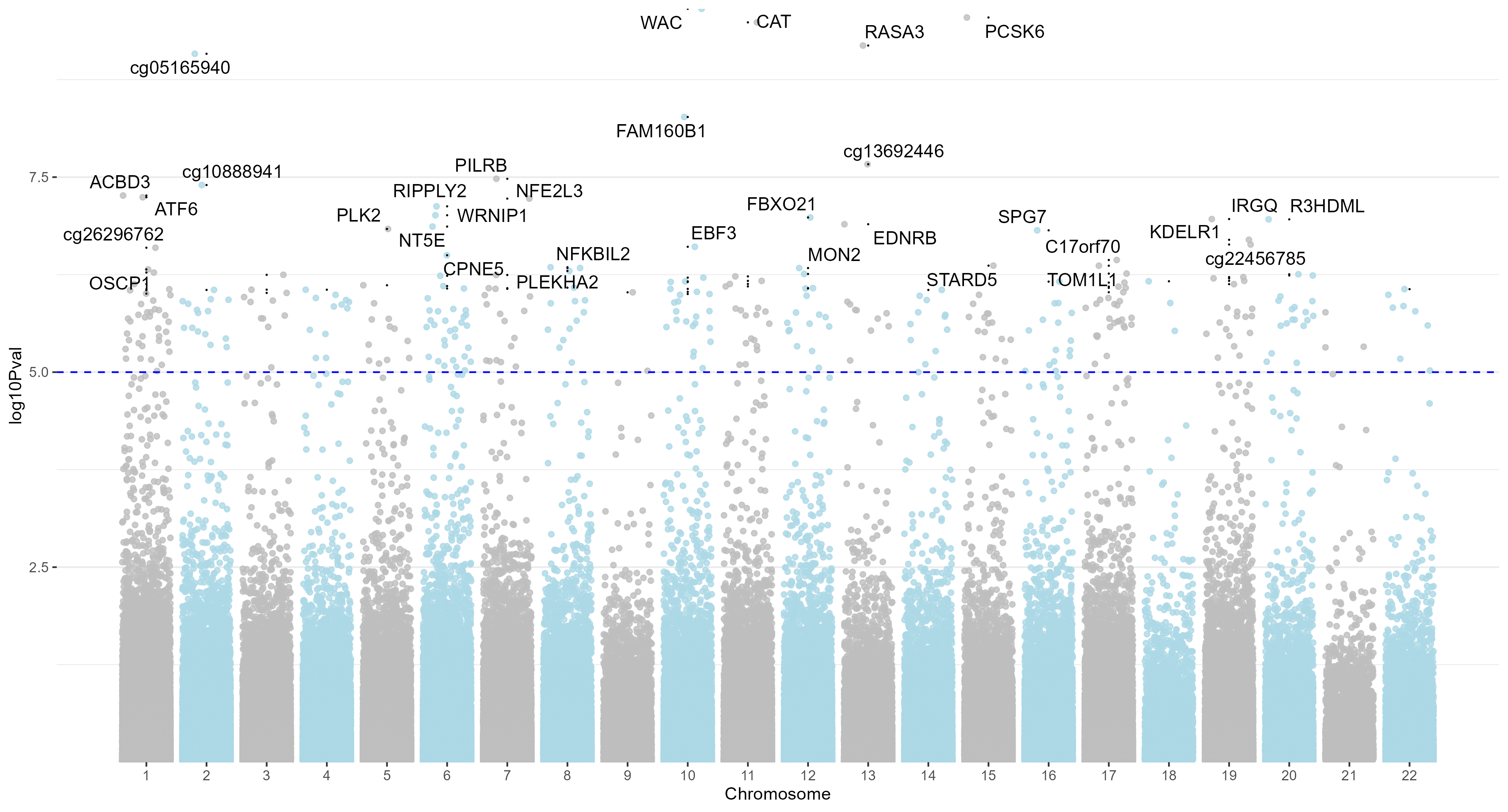

Supplement: Supplementary file 8 — Supplementary Fig. 3 [file 41380_2024_2752_MOESM8_ESM.tif]

Supplementary Figure 4

b)

a)


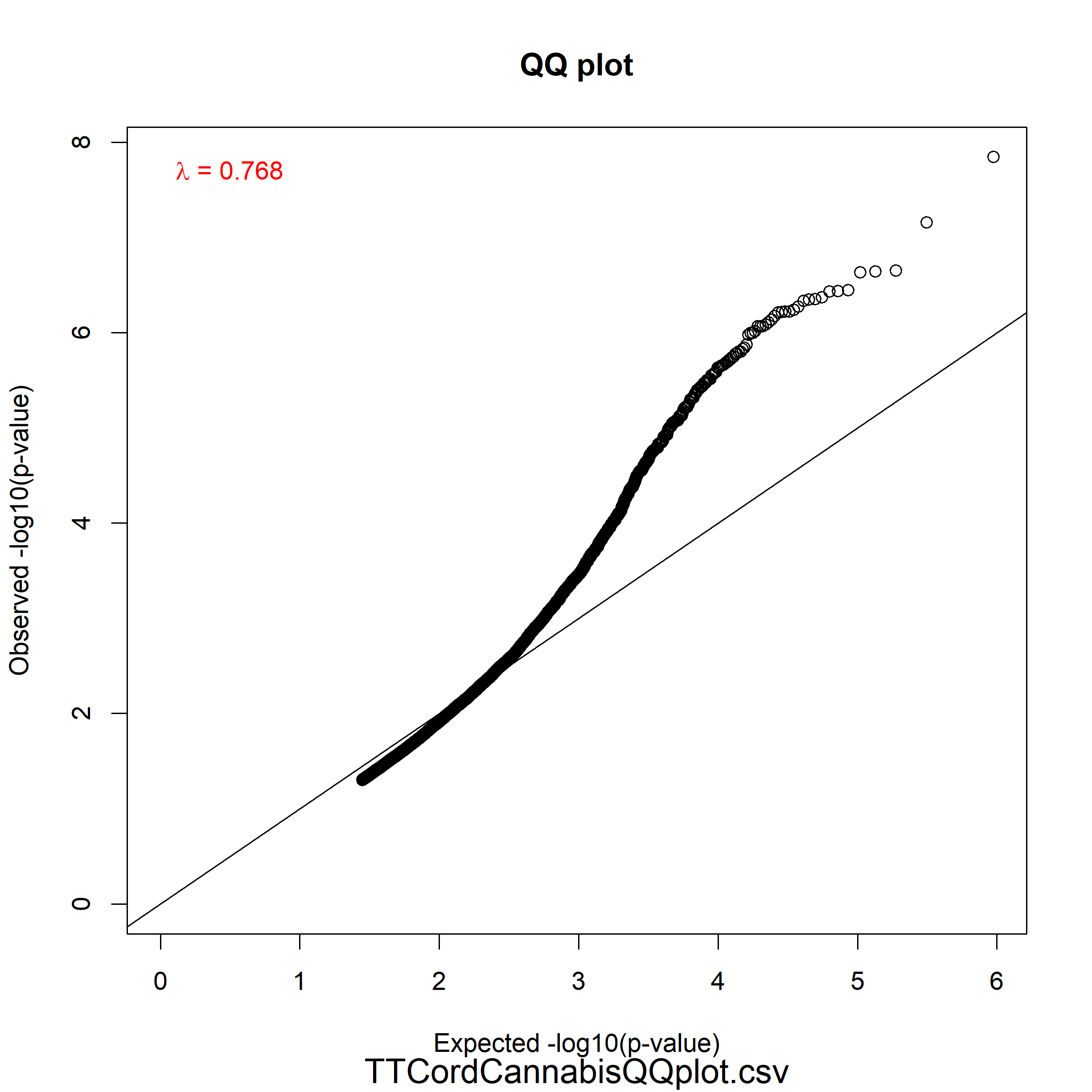

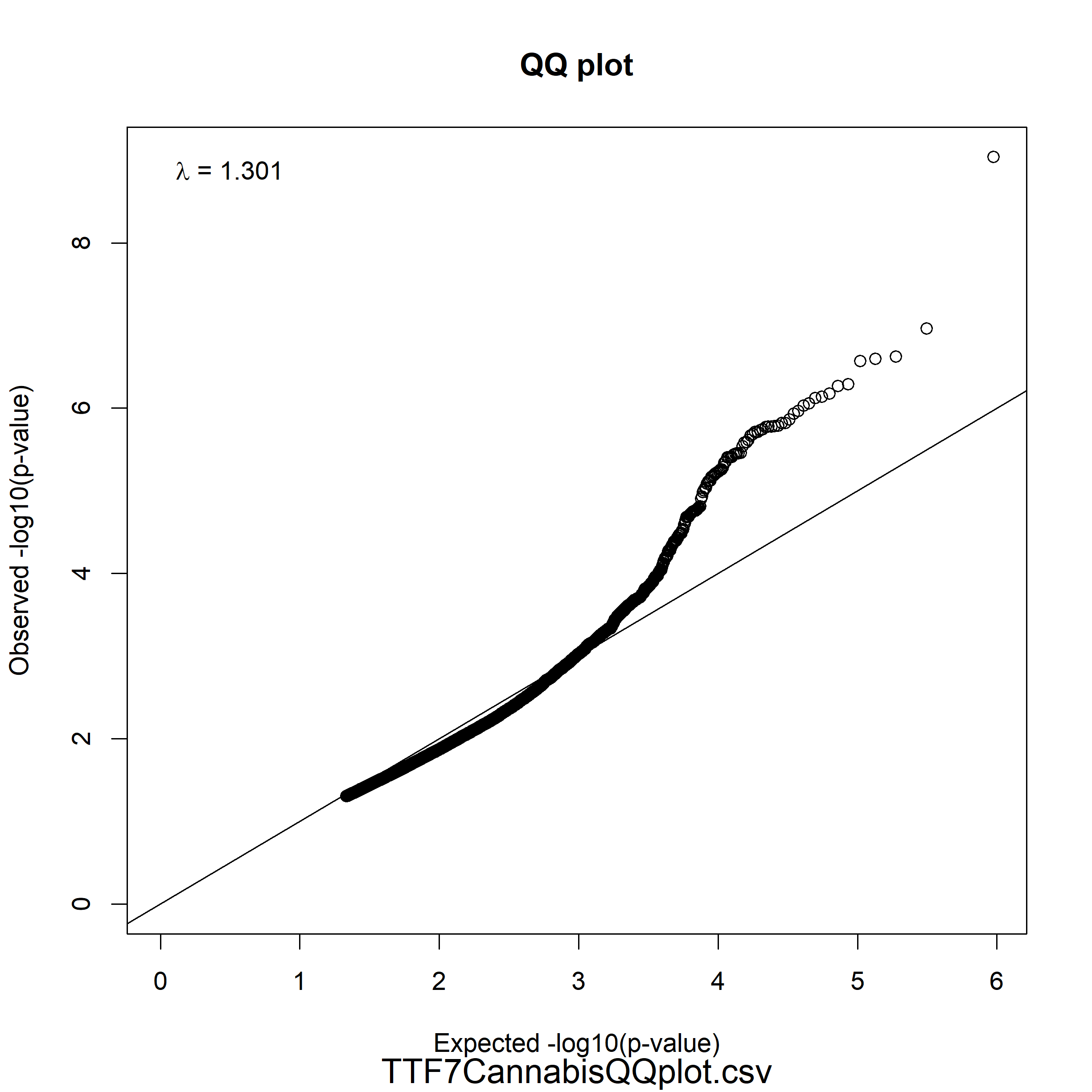


d)

c)


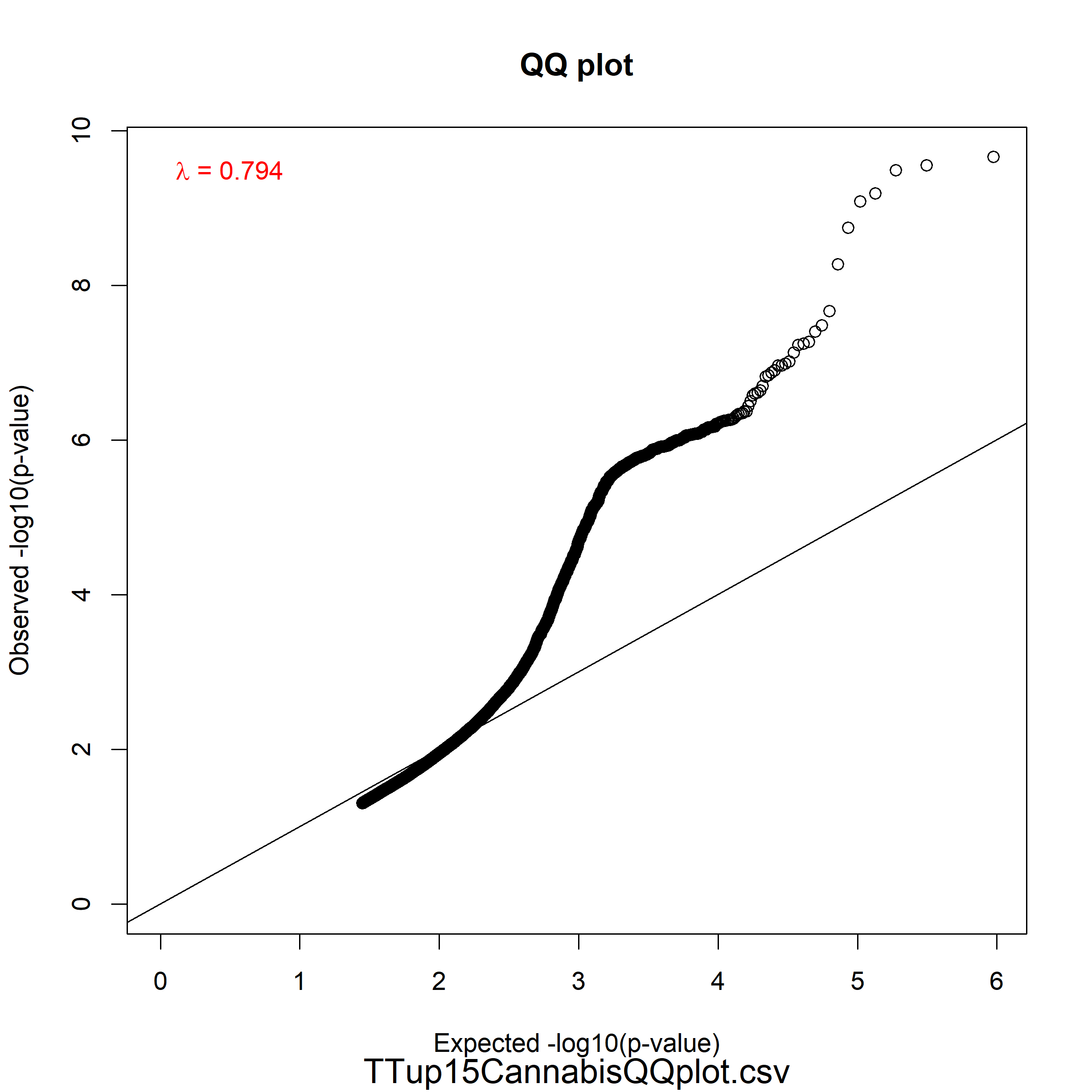

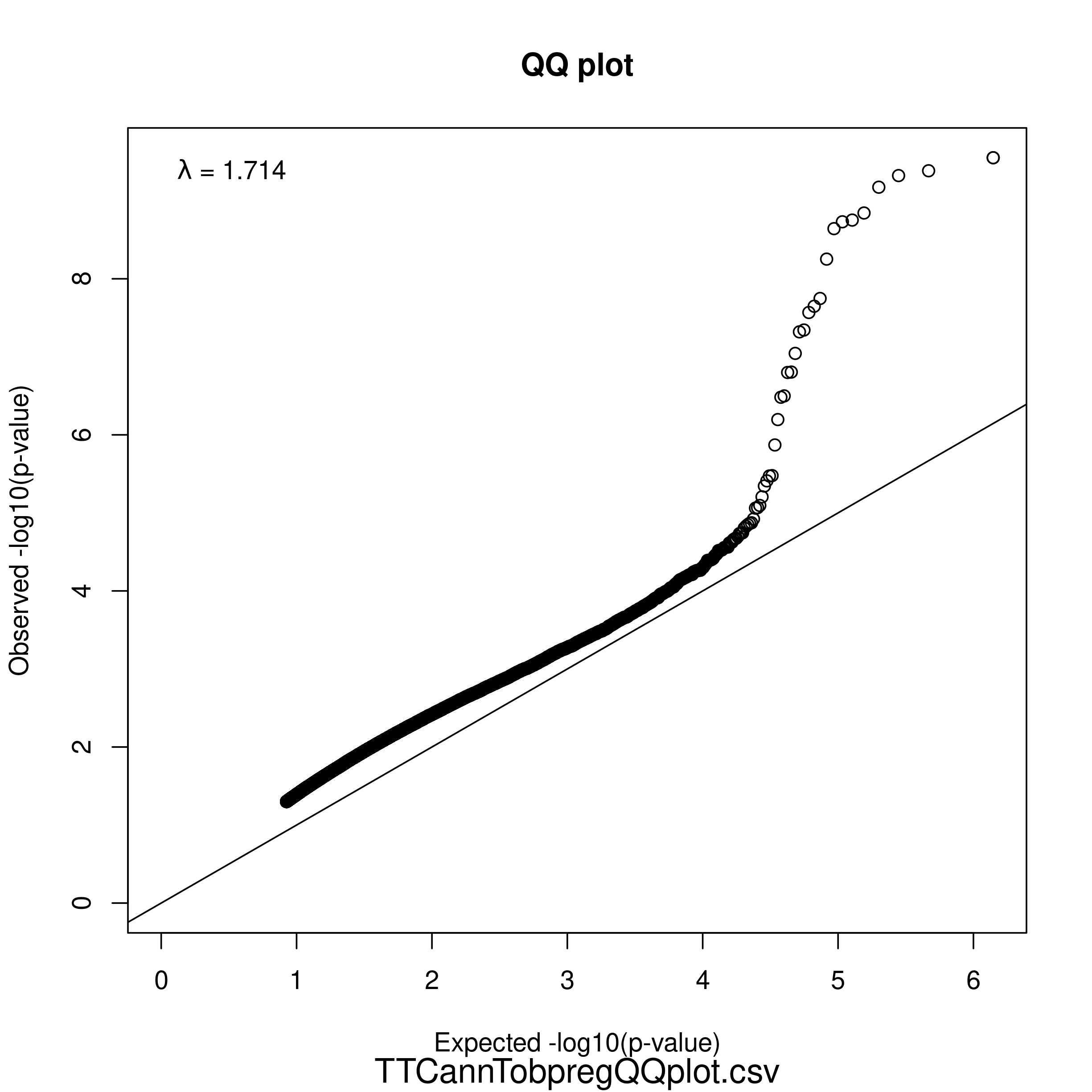


e)


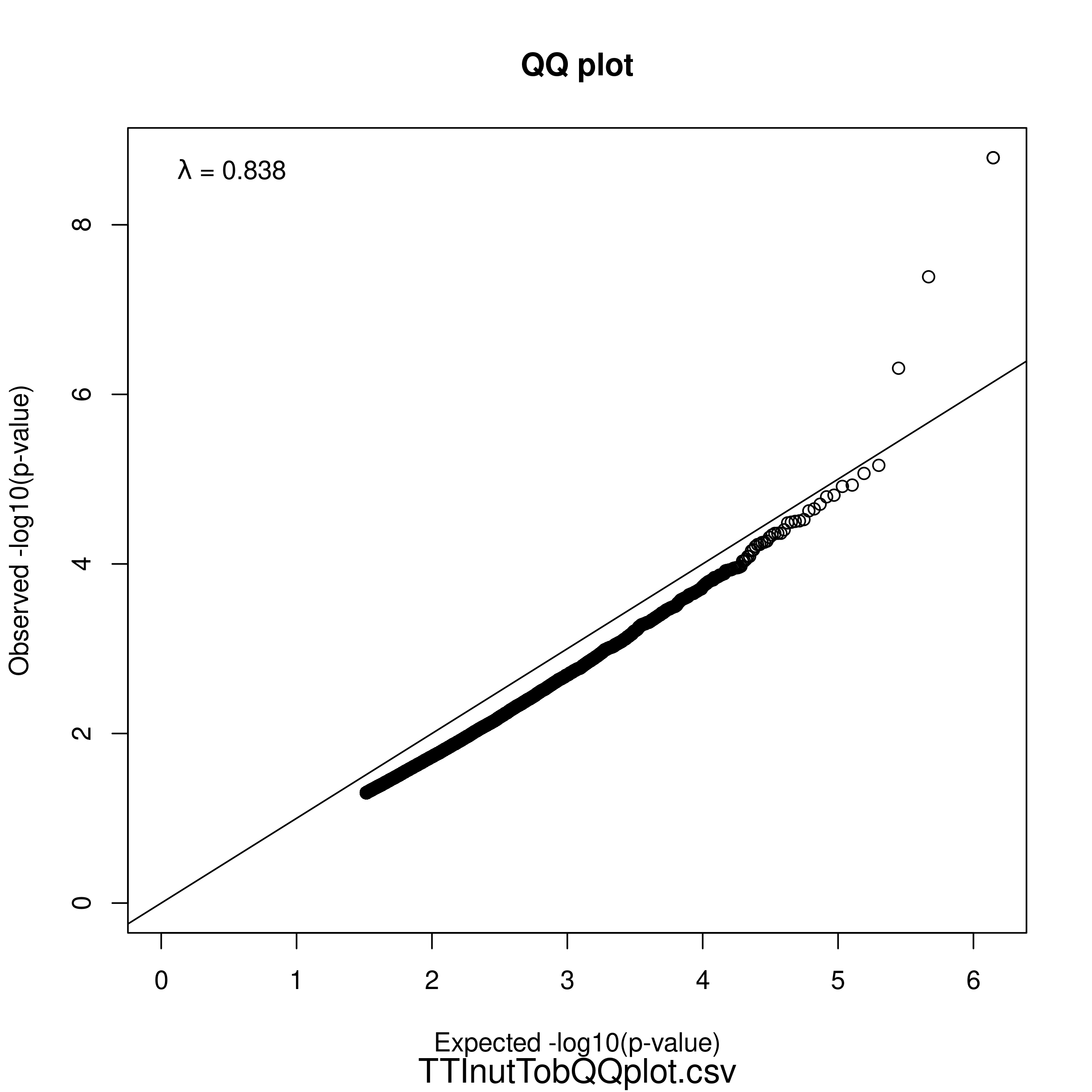

Supplement: Supplementary file 9 — Supplementary Fig. 4 [file 41380_2024_2752_MOESM9_ESM.docx]

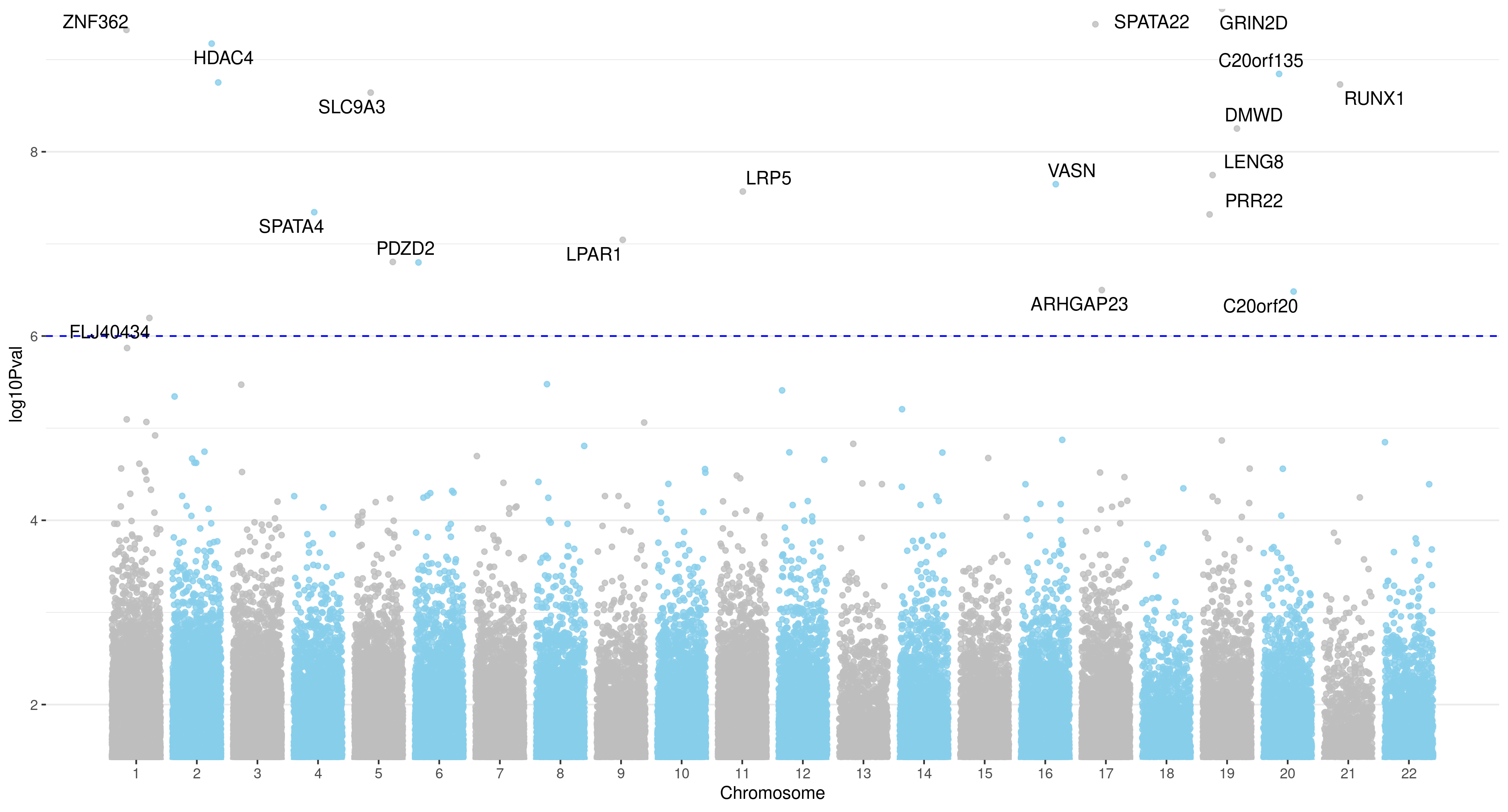

Supplement: Supplementary file 10 — Supplementary Fig. 5 [file 41380_2024_2752_MOESM10_ESM.tif]

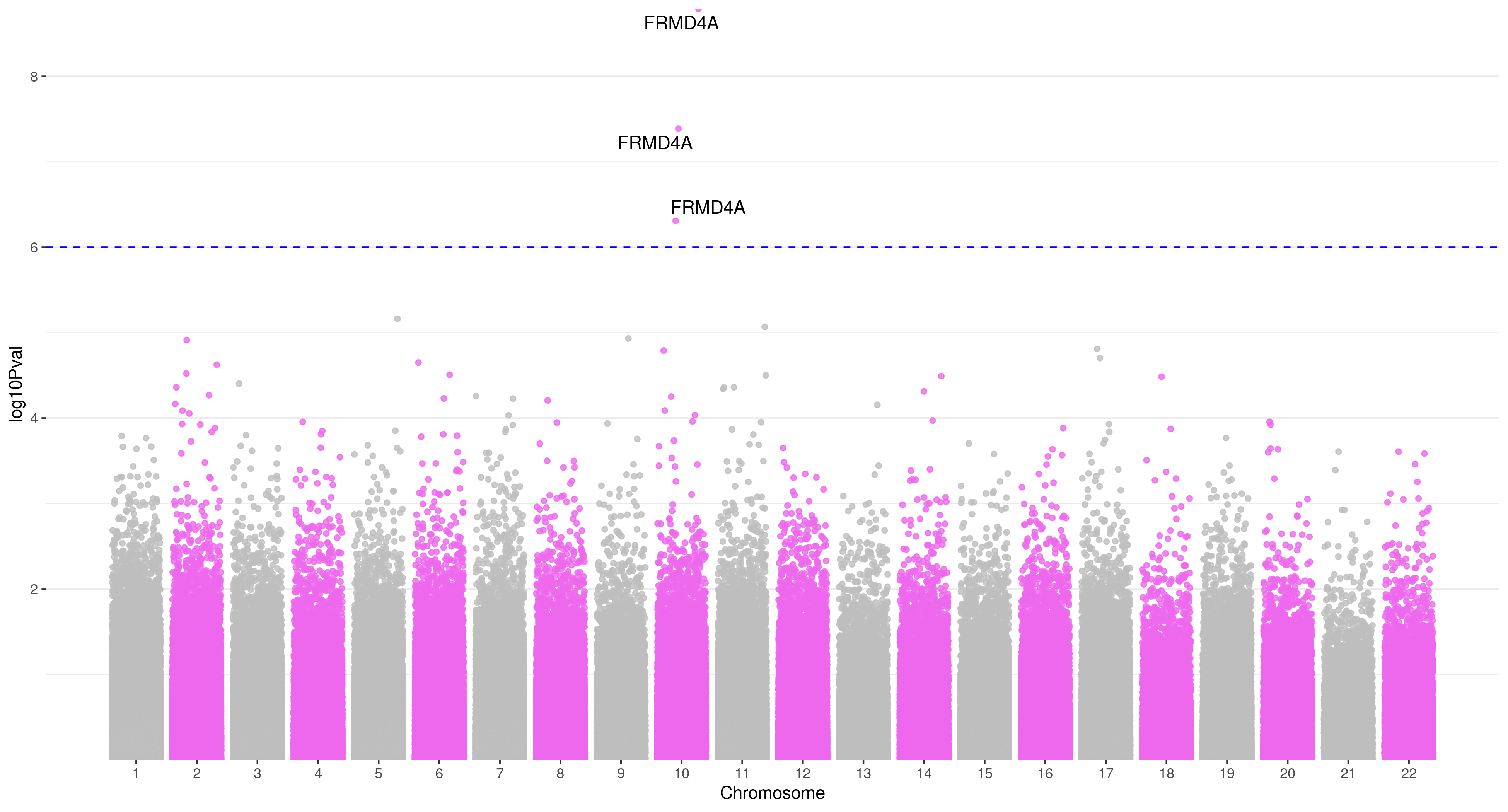

Supplement: Supplementary file 11 — Supplementary Fig. 6 [file 41380_2024_2752_MOESM11_ESM.tif]
